# Widely targeted metabolomics analysis of *Sanghuangporus vaninii* mycelia and fruiting bodies at different harvest stages

**DOI:** 10.3389/fmicb.2024.1391558

**Published:** 2024-05-23

**Authors:** Yue Qi, Xiao-Ying Guo, Xin-Yue Xu, Jian-Xuan Hou, Shi-Lai Liu, Hong-Bo Guo, Ai-Guo Xu, Rui-Heng Yang, Xiao-Dan Yu

**Affiliations:** ^1^College of Biological Science and Technology, Shenyang Agricultural University, Shenyang, China; ^2^College of Life Engineering, Shenyang Institute of Technology, Fushun, China; ^3^Alpine Fungarium, Tibet Plateau Institute of Biology, Lhasa, China; ^4^Institute of Edible Fungi, Shanghai Academy of Agricultural Sciences, Shanghai, China

**Keywords:** *Sanghuangporus vaninii*, mycelium, fruiting body, metabolomics, TCMSP

## Abstract

*Sanghuangprous vaninii* is a medicinal macrofungus cultivated extensively in China. Both the mycelia and fruiting bodies of *S. vaninii* have remarkable therapeutic properties, but it remains unclear whether the mycelia may serve as a substitute for the fruiting bodies. Furthermore, *S. vaninii* is a perennial fungus with therapeutic components that vary significantly depending on the growing year of the fruiting bodies. Hence, it is critical to select an appropriate harvest stage for *S. vaninii* fruiting bodies for a specific purpose. With the aid of Traditional Chinese Medicine Systems Pharmacology Database and Analysis Platform (TCMSP), metabolomics based on ultra-high performance liquid chromatography coupled to triple quadrupole mass spectrometry (UHPLC-QQQ-MS) was used to preliminarily determine 81 key active metabolites and 157 active pharmaceutical metabolites in *S. vaninii* responsible for resistance to the six major diseases. To evaluate the substitutability of the mycelia and fruiting bodies of *S. vaninii* and to select an appropriate harvest stage for the fruiting bodies of *S. vaninii*, we analyzed the metabolite differences, especially active metabolite differences, among the mycelia and fruiting bodies during three different harvest stages (1-year-old, 2-year-old, and 3-year-old). Moreover, we also determined the most prominent and crucial metabolites in each sample of *S. vaninii*. These results suggested that the mycelia show promise as a substitute for the fruiting bodies of *S. vaninii* and that extending the growth year does not necessarily lead to higher accumulation levels of active metabolites in the *S. vaninii* fruiting bodies. This study provided a theoretical basis for developing and using *S. vaninii*.

## 1 Introduction

*Sanghuangporus vaninii* (Ljub.) L.W. Zhou & Y.C. Dai is a medicinal and edible homologous macrofungus well-known as “forest gold.” It is used as a herbal remedy and healthy food in Asian countries. Modern research has demonstrated that *S. vaninii* has excellent medicinal values, such as antitumor/cancer (Jia et al., 2018; Wan et al., 2020, 2022; Guo et al., 2022; Qiu et al., 2022), antioxidant (Lin et al., 2017; Xu et al., 2017; Wang et al., 2023), antidiabetic (Huang et al., 2022a,b,c), anti-inflammatory (Lin et al., 2017; Hou et al., 2022), antiproliferative (Lin et al., 2017), anti-Parkinsonism (Li et al., 2021), and anti-gout (Guo et al., 2021). Polysaccharides, flavonoids, terpenoids, and phenolic compounds are mainly responsible for these biological activities. Over the past few years, investigation on bioactive monomers of *S. vaninii* has been performed less and was primarily focused on polysaccharides (Lin et al., 2017; Guo et al., 2022; Huang et al., 2022a,b,c) and styrylpyrones (Jia et al., 2018). The key bioactive components with health-promoting functions in *S. vaninii* have not been designated (Ru et al., 2014; Ishara et al., 2022). For better exploitation and application of *S. vaninii*, it is necessary to thoroughly identify their functional compounds. Traditional Chinese Medicine Systems Pharmacology Database And Analysis Platform (TCMSP) was built based on the framework of systems pharmacology for herbal medicines (Zhou et al., 2022). TCMSP provides drug targets and their corresponding diseases for each active compound, and it also lists 12 important ADME-related properties such as oral bioavailability (OB) and drug-likeness (DL) (Zhou et al., 2022). Widely targeted metabolomics integrates the advantages of non-target and targeted metabolite detection technologies, achieving high throughput and exhibiting high sensitivity and comprehensive coverage (Chu et al., 2020). In recent years, metabolomics has been successfully and widely applied to medicinal mushrooms (Bhardwaj et al., 2017; Jia et al., 2018; Oh et al., 2019; Deng et al., 2021; Dong et al., 2022). In this study, integrated TCMSP and metabolomics technologies were used as a tool for capturing a comprehensive profile of active compounds in *S. vaninii*.

Due to the wide range of bioactivities of *S. vaninii*, the commercialization of its products is increasing rapidly, leading to a substantial demand for this rare fungus (Zhou et al., 2022). The shortage of available basidiocarps of *S. vaninii* is one of the most important limitations for the utilization of *S. vaninii* (Zhou et al., 2022). As the production cycle of the mycelium is considerably shortened compared with that of fruiting bodies, obtaining the mycelium is easier than the fruiting bodies of *S. vaninii*. Therefore, increased efforts for investigating the possibility of replacing the fruiting bodies with the mycelium of *S. vaninii* in the process of production are essential for promoting the exploitation and utilization of *S. vaninii*. Extensive research carried out, mainly in China, indicated high nutrient composition and content and biological activities of the mycelium of “Sanghuang,” and the results suggested that fermented mycelia seem feasible to replace the fruiting bodies (Ye et al., 2019; Zhang et al., 2020). Ye et al. (2019) analyzed the nutrient composition and the antineoplastic activity of the main functional compounds in the fruiting body and mycelium of *Phellinus igniarius*, and the results showed that they had a similar nutrient composition, and the mycelia also have strong antitumor potential against SW620 and HepG2 tumor cells. Zhang et al. (2020) analyzed the metabolite composition and content, as well as antioxidant and antineoplastic activities in the mycelium and fruiting body of *Sanghuangporous sanghuang*, and the results showed that the antioxidant activity of the mycelium was higher than that of the fruiting body, which has a significant correlation with the total flavonoid content. To the best of our knowledge, no information is available about the metabolic differences between the mycelium and the fruiting body of *S. vaninii*. In this study, the fruiting body and mycelium of *S. vaninii* were evaluated for the composition, content, and activities of the main functional compounds using metabolomics, aiming to lay a foundation for further exploitation and utilization of *S. vaninii*.

*Sanghuangporus vaninii* is a major cultivar with a high, stable yield in numerous provinces of China (Ye et al., 2019). It has reached the mainstream market in China as a drug with excellent antitumor potential (Ye et al., 2019). The quality of the fruiting bodies of *S. vaninii* mainly depends on the content of active components, which is closely related to the harvest time (He et al., 2021; Zhang et al., 2020). Metabolomics approaches have been successfully applied to medicinal mushrooms at different developmental stages (Satria et al., 2019). This study also examined the metabolic variations among the fruiting bodies of *S. vaninii* at three harvest stages (1-year-old, 2-year-old, and 3-year-old). Furthermore, we conducted a thorough screening process to identify potential biomarkers and prominent metabolites that are closely linked to health benefits. This study not only identified novel metabolites with health-promoting values for humans in *S. vaninii* but also helped enhance people’s understanding of metabolic differences between the mycelium and fruiting bodies of *S. vaninii* at the three harvest stages.

## 2 Materials and methods

### 2.1 Sample preparation and extraction

*S. vaninii* fruiting body samples were harvested at 1, 2, and 3 years during March 2022. *S. vaninii* fruiting bodies were collected three times every growth year. These fruiting bodies were cultivated and provided by Fushun Rencongzhong Ecological Mushroom Industry Co., LTD. *S. vaninii* mycelium samples were acquired by incubating the sample for 14 days in the potato dextrose agar (PDA) medium, filtered by a three-layer gauze, and repeatedly cleaned in a sterile chamber. The identity of each sample, based on morphological and molecular evidence, was confirmed as *S. vaninii*. The abbreviations and related information of the samples are presented in Table 1. Three replicate samples were collected from the pileus for each sample. The harvested materials were weighed, immediately frozen in liquid nitrogen, and stored at −80°C until use. These samples were freeze-dried by a vacuum freeze-dryer (Scientz-100F). The lyophilized samples were crushed by using a mixer mill (MM 400, Retsch) with a zirconia bead for 1.5 min at 30 Hz. A total of 50 mg lyophilized powder was weighed and extracted at 4°C with 1.2 mL of 70% aqueous methanol solution.

**Table 1:** The abbreviations and detailed information of the samples of *S. vaninii*.

Sample abbreviation	Sample full name	Production area	Growth pattern
SVM	Mycelium of *Sanghuangporus vaninii*	Shenyang City, Liaoning Province, China	PDA medium
SVF	Fruiting bodies of *Sanghuangporus vaninii*	Fushun City, Liaoning Province, China	Cut-log cultivation
SVI	1-year-old fruiting bodies of *Sanghuangporus vaninii*		
SVII	2-year-old fruiting bodies of *Sanghuangporus vaninii*		
SVIII	3-year-old fruiting bodies of *Sanghuangporus vaninii*		

### 2.2 UPLC conditions

The sample extracts were analyzed using a UPLC-ESI-MS/MS system (UPLC, SHIMADZU Nexera X2^1^; MS, Applied Biosystems 4500Q TRAP^2^). The analytical column was Agilent SB-C18 (1.8 μm, 2.1 mm × 100 mm), and the mobile phase comprised solvent A, pure water with 0.1% formic acid, and solvent B, acetonitrile with 0.1% formic acid. Sample measurements were performed with a gradient program that employed the starting conditions of 95% A, 5% B, linear gradient to 5% A and 95% B within 9 min, 5% A and 95% B for 1 min, 95% A and 5.0% B within 1.1 min, and kept for 2.9 min. The flow rate was 0.35 mL/min, the column oven temperature was 40°C, and the injection volume was 4 μL. The effluent was alternatively connected to an ESI-triple quadrupole (QqQ)-linear ion trap (QTRAP)-MS.

### 2.3 ESI-Q TRAP-MS/MS

The ESI source operation parameters were as follows: source temperature, 500°C; ion spray voltage (IS), 5,500 V (positive ion mode)/−4,500 V (negative ion mode); ion source gas I (GSI), gas II (GSII), and curtain gas were 50, 60, and 25 psi, respectively; and collision-activated dissociation, high. QqQ scans were acquired under multiple reaction monitoring (MRM), with the collision gas (nitrogen) set to medium. Declustering potential (DP) and collision energy (CE) analyses for individual MRM transitions were performed, with further DP and CE optimization. A specific set of MRM transitions was monitored for each period according to the metabolites eluted within this period.

### 2.4 Qualitative and quantitative analyses of metabolites

Crucial MS parameters, such as DP, CE, retention time (RT), Q1 (precursor ion), and Q3 (product ion), were used to identify metabolites from the Metware database (Wuhan Metware Biotechnology Co. Ltd., Massachusetts, United States). After identifying the compounds, we conducted a comparative analysis against the database and classified them into Class I and Class II based on their structural characteristics. Class I represents the primary classification of compounds, while Class II provides a more detailed categorization of metabolites in Class I. Metabolites were quantified using the MRM mode for mass spectrometry of metabolites.

### 2.5 Identification of key active metabolites and active pharmaceutical metabolites in *Sanghuangporus vaninii* for resistance to the six major diseases

To identify the key active metabolites, metabolites detected through UHPLC-QQQ-MS analysis were queried under the TCMSP database (Ru et al., 2014). The retrieved chemical compositions were further used to determine the key active metabolites. Using the screening criterion of oral bioavailability (OB) ≥ 5% and drug-likeness (DL) ≥ 0.14, the key active metabolites in *S. vaninii* were determined, as described previously (Li et al., 2021). To determine the active metabolites for tumor/cancer disease resistance, the determined metabolites were queried in the CancerHSP database under the TCMSP analysis platform. The retrieved metabolites were regarded as the antitumor/anticancer metabolites. Furthermore, the keywords “diabetes,” “cardiovascular disease,” “Parkinson,” “Alzheimer,” and “asthma” were used to query under the TCMSP database. Finally, the active pharmaceutical metabolites for resistance to the six major diseases in *S. vaninii* were determined, as described previously (Li et al., 2021).

### 2.6 Data analysis

Unsupervised principal component analysis (PCA) was performed by statistics function prcomp within R. The data were unit variance-scaled before performing unsupervised PCA. Hierarchical cluster analysis (HCA) of normalized signal intensities between SVM, SVI, SVII, and SVIII, as well as their replicates, was computed in R and presented as heatmaps. In differential metabolite analysis, differential metabolites between two groups were determined based on the scores of a value importance plot (VIP) ≥ 1 and |Log_2_fold change (FC)| ≥ 1.0. VIP values were extracted from orthogonal projections to latent structures-discriminant analysis (OPLS-DA) results, which also contain score plots and permutation plots, generated using the R package MetaboAnalystR. The data were log-transformed and mean-centered before performing OPLS-DA. To avoid overfitting, a permutation test (200 permutations) was used.

## 3 Results

### 3.1 Detection of metabolites in *Sanghuangporus vaninii*

**Figure 1:**
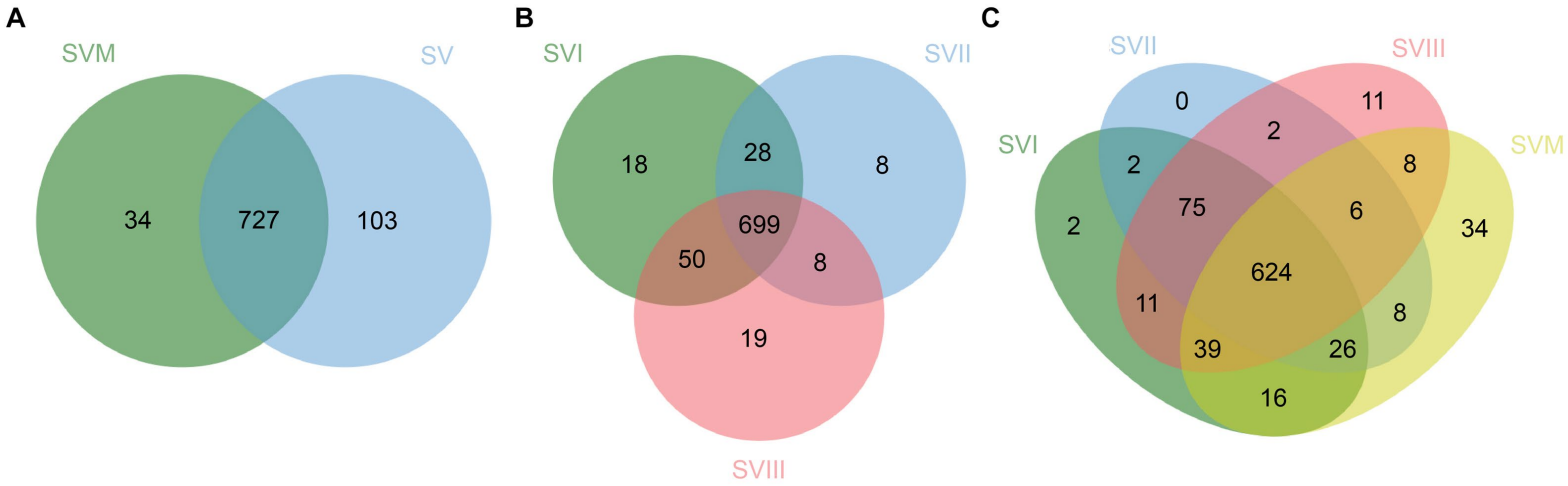
Venn diagrams for the number of metabolites in four samples of *S. vaninii*. **(A)** SVM vs. SVF; **(B)** SVI vs. SVII vs. SVIII; **(C)** SVM vs. SVI vs. SVII vs. SVIII.

Profiling of extracts of four samples of *S. vaninii* through UHPLC-QQQ-MS analysis resulted in the detection of 864 metabolites, including 143 lipids, 113 phenolic acids, 113 organic acids, 99 amino acids and derivatives, 97 flavonoids, 78 alkaloids, 77 carbohydrates, 64 nucleotides and derivatives, 37 terpenoids, 14 coumarins and lignans, 12 organic acids, 11 vitamins, 3 chromones, and 3 quinones. The detailed information is listed in Supplementary Table S1. Furthermore, there were a total of 727 metabolites present in both SVF and SV. In addition, 34 metabolites were exclusively present in SVM, whereas 103 metabolites were exclusively present in SVF (Figure 1A). The information on the unique metabolites in SVM/SVF is listed in Supplementary Table S2. Furthermore, there were a total of 699 metabolites present in SVI, SVII, and SVIII. Out of these, 18 metabolites were exclusively present in SVI, eight metabolites were exclusively present in SVII, and 19 metabolites were exclusively present in SVIII (Figure 1B). The information on the unique metabolites in SVI/SVII/SVIII is listed in Supplementary Table S3. A total of 624 metabolites were present in SVM, SVI, SVII, and SVIII. Among them, 2 metabolites were exclusively present in SVI, 11 metabolites were exclusively present in SVIII, and 34 metabolites were exclusively present in SVM (Figure 1C).

### 3.2 Identification of the key active metabolites in *Sanghuangporus vaninii*

To identify the key active metabolites in *S. vaninii*, we performed a query of the detected 864 metabolites under the TCMSP database. Consequently, the chemical composition of 253 out of the 864 metabolites was retrieved. Using the screening criterion of OB ≥ 5% and DL ≥ 0.14%, a total of 79 metabolites were identified as the key active metabolites. Two well-known styrylpyrone-class compounds, namely, hispolon and hispidin, have been widely isolated and verified to possess significant biological properties in *Sanghuangporus* species (Gonindard et al., 1997; Anouar et al., 2014; Wu et al., 2014; Benarous et al., 2021; Liao et al., 2021; Palkina et al., 2021; Saka et al., 2023). However, these two compounds have not been included in the TCMSP database. In conclusion, a total of 81 metabolites were determined as the key active metabolites in *S. vaninii*, which are mainly involved in flavonoids (30), lipids (18), and terpenoids (15). The key active metabolites in *S. vaninii* are listed in Supplementary Table S4. Out of the 81 key active metabolites, 51 metabolites were relevant to at least 136 diseases, mainly including cancers/tumors, cardiovascular disease, chronic inflammatory diseases, and Alzheimer’s disease. The remaining 30 metabolites have no corresponding target proteins and diseases, but 14 of these metabolites exhibited very high DL values (DL ≥ 0.72). The use of the 14 metabolites might potentially contribute to the advancement of novel pharmaceuticals (Li et al., 2021). These metabolites are betulin, madasiatic acid, lupenone, alphitolic acid, isomangiferolic acid, mangiferolic acid, 3-epiursolic acid, asiatic acid, pomolic acid, hederagenin, limonin, betulinic acid, 2-hydroxyoleanolic acid, dehydroabietic acid, hesperetin-5-O-glucoside, and hesperetin-7-O-glucoside (Supplementary Table S4).

### 3.3 Identification of active pharmacological metabolites in *Sanghuangporus vaninii* associated with resistance to the six major diseases

Cancers/tumors, cardiovascular disease, diabetes, asthma, Parkinson’s disease, and Alzheimer’s disease are six major threats to the health of humans around the world. To identify the active pharmacological metabolites associated with resistance to the six major diseases in *S. vaninii*, we performed a query of the detected 864 metabolites under the TCMSP database. As a result, a total of 155 metabolites were identified as active pharmacological metabolites, which were resistant to at least one disease mentioned above. In addition to hispolon and hispidin discussed above, a total of 157 metabolites were determined as active pharmacological metabolites in *S. vaninii* for resistance to the six major diseases, mainly including phenolic acids (46) and flavonoids (30). These active pharmacological metabolites for resistance to the six major diseases are listed in Supplementary Table S5. It is noteworthy that some of these metabolites corresponded to resistance to numerous diseases as mentioned above. For example, diisobutyl phthalate, cinnamic acid, protocatechuic acid, 3-(3-hydroxyphenyl)-propionic acid, sinensetin, hispidulin, tangeretin, D-(−)-mandelic acid, phenyl pyruvic acid, DL-3-phenyllactic acid, erucic acid, elaidic acid, and dehydroabietic acid corresponded to all the six major diseases mentioned above (Supplementary Table S5). These metabolites may play the most pivotal role in the pharmacological treatment of the six main diseases (Li et al., 2021).

Among the 157 active pharmacological metabolites in *S. vaninii* associated with resistance to the six major diseases, 52 metabolites were also identified as the key active metabolites. These 52 metabolites consisted of 25 flavonoids (rhamnocitrin, hyperin, quercetagetin, narcissin, nobiletin, sinensetin, tangeretin, isosinensetin, hispidulin, cosmosiin, diosmin, galangin, butin, narirutin, hesperidin, sakuranin, naringin, prunin, eriodictyol, astilbin, gancaonin G, prunetin, isohyperoside, catechin, and 3,5,6,7,8,3′,4′-heptamethoxyflavone), 14 lipids (ricinoleic acid, methyl linolenate, linoleic acid, punicic acid, γ-linolenic acid, elaidic acid, vaccenic acid, petroselinic acid, erucic acid, eicosenoic acid, arachidic acid, arachidonic acid, 9-hydroxy-10,12,15-octadecatrienoic acid, and 1-linoleoylglycerol), 5 phenolic acids (hispolon, hispidin, chlorogenic acid, usnic acid, and diisooctyl phthalate), 2 terpenoids (ursolic acid and dehydroabietic acid), 2 saccharides (D-sucrose and raffinose), 2 vitamins (vitamin B2 and vitamin K1), 1 nucleotide and derivative (uridine 5′-monophosphate), and one alkaloid (betaine). These metabolites were identified as both key active metabolites and active pharmacological metabolites in *S. vaninii* associated with resistance to the six major diseases and might be the most crucial metabolites with biological activities in *S. vaninii* (Li et al., 2021).

### 3.4 Multivariate analysis

**Figure 2:**
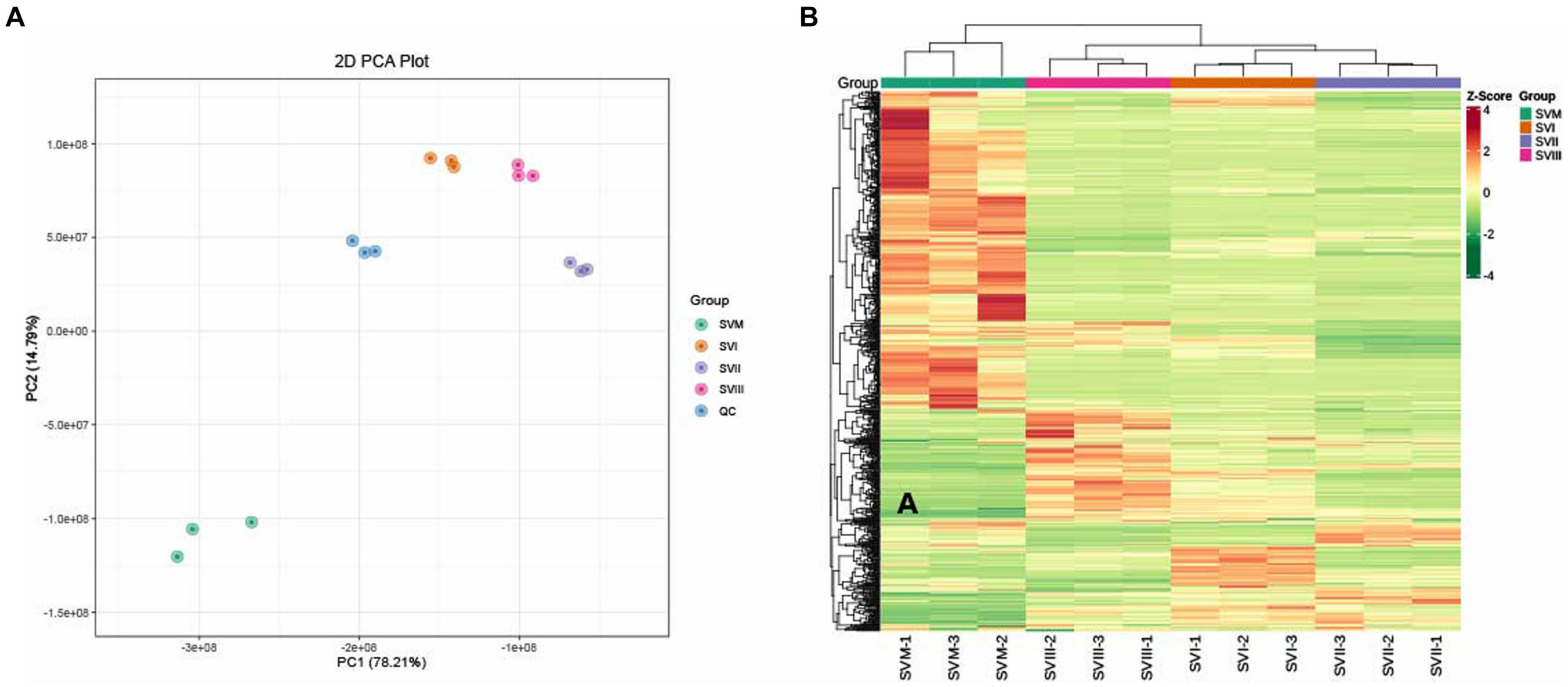
PCA **(A)** and HCA **(B)** analyses of metabolites identified in the SVM, SVI, SVII, and SVIII. The colors of the heatmap indicated the relative content of each metabolite, from low (green) to high (red).

Multivariate statistical analysis was performed to assess the metabolic differences among SVM, SVI, SVII, and SVIII. In the PCA diagram (Figure 2A), significant segregation occurred among the four samples. The QC sample is a mixture of all sample extracts, which were projected to the same area, thus the analysis was stable and repeatable. The HCA diagram (Figure 2B) also showed significant differences among the four samples. The results exhibited clustering across three biological replicates, suggesting strong homogeneity and data reliability.

### 3.5 Differential metabolite analysis

**Figure 3:**
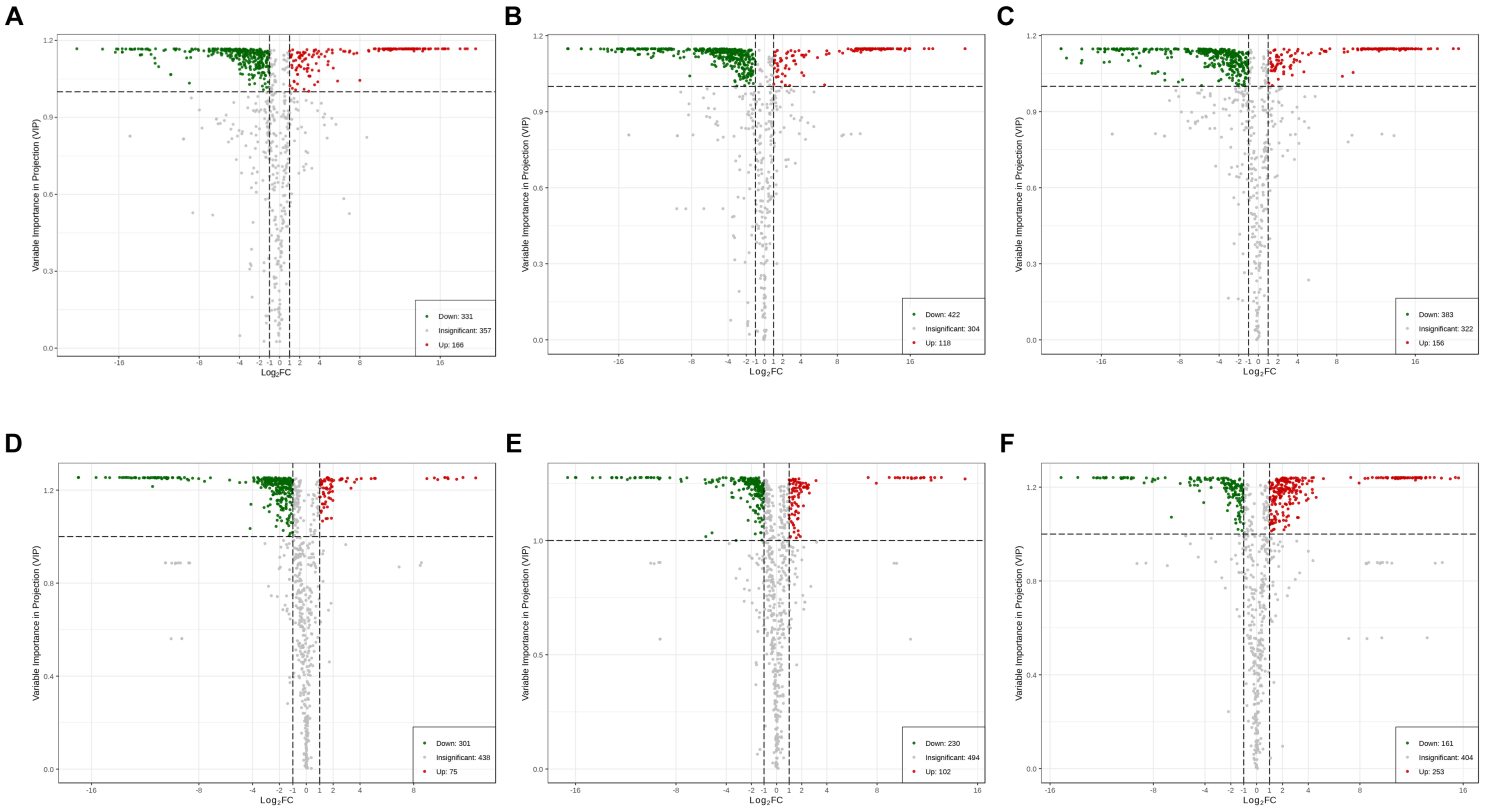
Volcano plots showing the number of differential metabolites for each comparison group. **(A)** SVM vs. SVI; **(B)** SVM vs. SVII; **(C)** SVM vs. SVIII; **(D)** SVI vs. SVII; **(E)** SVI vs. SVIII; **(F)** SVII vs. SVIII.

To explore the metabolic differences between SVI, SVII, SVIII, and SVM, a pairwise comparison was conducted based on the VIP value ≥ 1 and |Log_2_fold change (FC)| ≥ 1. When comparing SVM with SVF, there were 497 significantly differential metabolites between SVM and SVI (166 upregulated and 331 downregulated) (Figure 3A); there were 540 significantly differential metabolites between SVM and SVII (118 upregulated and 422 downregulated) (Figure 3B); and there were 539 significantly differential metabolites between SVM and SVIII (156 upregulated and 383 downregulated) (Figure 3C). When comparing SVI, SVII, and SVIII, there were 376 significantly differential metabolites between SVI and SVII (75 upregulated and 301 downregulated) (Figure 3D); there were 332 significantly differential metabolites between SVI and SVIII (102 upregulated and 230 downregulated) (Figure 3E); and there were 414 significantly differential metabolites between SVII and SVIII (253 upregulated and 161 downregulated) (Figure 3F). The number of differential metabolites based on the compound class in each pairwise comparison group is listed in Supplementary Table S6.

**Figure 4:**
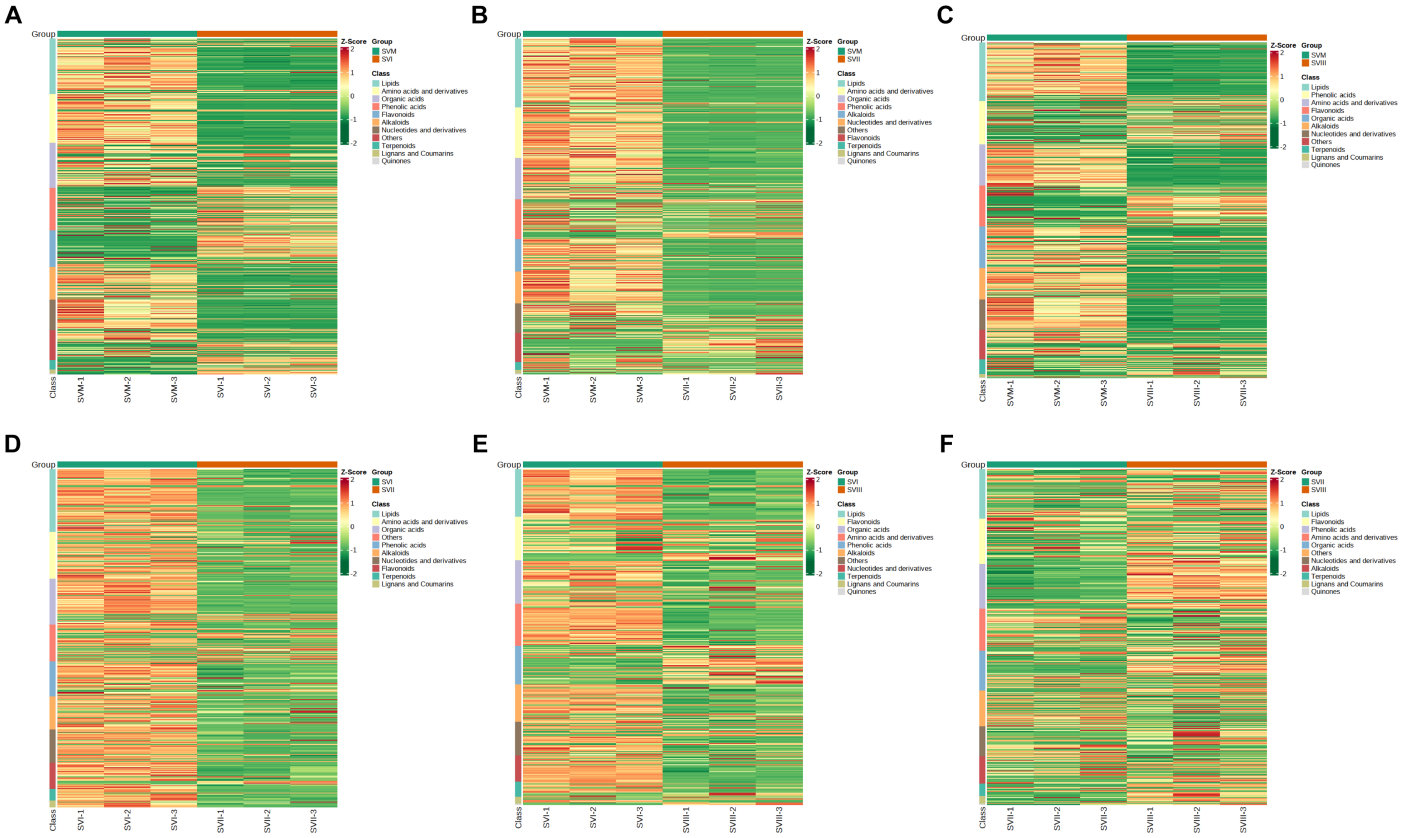
Heatmaps showing the expression levels of each differential metabolite for each comparison group. **(A)** SVM vs. SVI; **(B)** SVM vs. SVII; **(C)** SVM vs. SVIII; **(D)** SVI vs. SVII; **(E)** SVI vs. SVIII; **(F)** SVII vs. SVIII.

Comparing SVM and SVI, SVM was richer in lipids, amino acids and derivatives, nucleotides and derivatives, organic acids, and alkaloids, whereas SVI was richer in phenolic acids, flavonoids, and terpenoids (Figure 4A). Comparing SVM and SVII, SVM was richer in lipids, amino acids and derivatives, nucleotides and derivatives, organic acids, and alkaloids, whereas SVII was richer in phenolic acids and flavonoids (Figure 4B). Comparing SVM and SVIII, SVM was richer in lipids, amino acids and derivatives, nucleotides and derivatives, organic acids, and alkaloids, whereas SVIII was richer in phenolic acids and flavonoids (Figure 4C). Comparing SVI and SVII, almost all metabolites showed higher accumulation levels in SVI (Figure 4D). For SVI and SVIII, SVI was richer in lipids, amino acids and derivatives, nucleotides and derivatives, organic acids, alkaloids, and terpenoids, whereas SVIII was richer in phenolic acids and flavonoids (Figure 4E). For SVII and SVIII, SVII was richer in amino acids and derivatives, whereas SVIII was richer in lipids, phenolic acids, and organic acids (Figure 4F). The volcano plots (Figure 3) showed the number of differential metabolites, and the heatmap (Figure 4) showed the relative content of each metabolite among four samples.

### 3.6 Identification of potential biomarkers

#### 3.6.1 Identification of potential biomarkers of the mycelium and fruiting bodies of *Sanghuangporus vaninii*

A “biomarker” is an indicator of a bodily function that can be objectively measured (Spiller, 2011). In this study, the term “biomarker” refers to a discriminating indication of four different samples of *S. vaninii* (Li et al., 2021). Specifically, it refers to metabolites that show a considerably greater concentration (at least twice as much) in one sample compared to the other samples of *S. vaninii*. The use of biomarkers might offer a novel and effective alternative method for quickly and simply identifying the fruiting bodies of different growth years.

**Figure 5:**
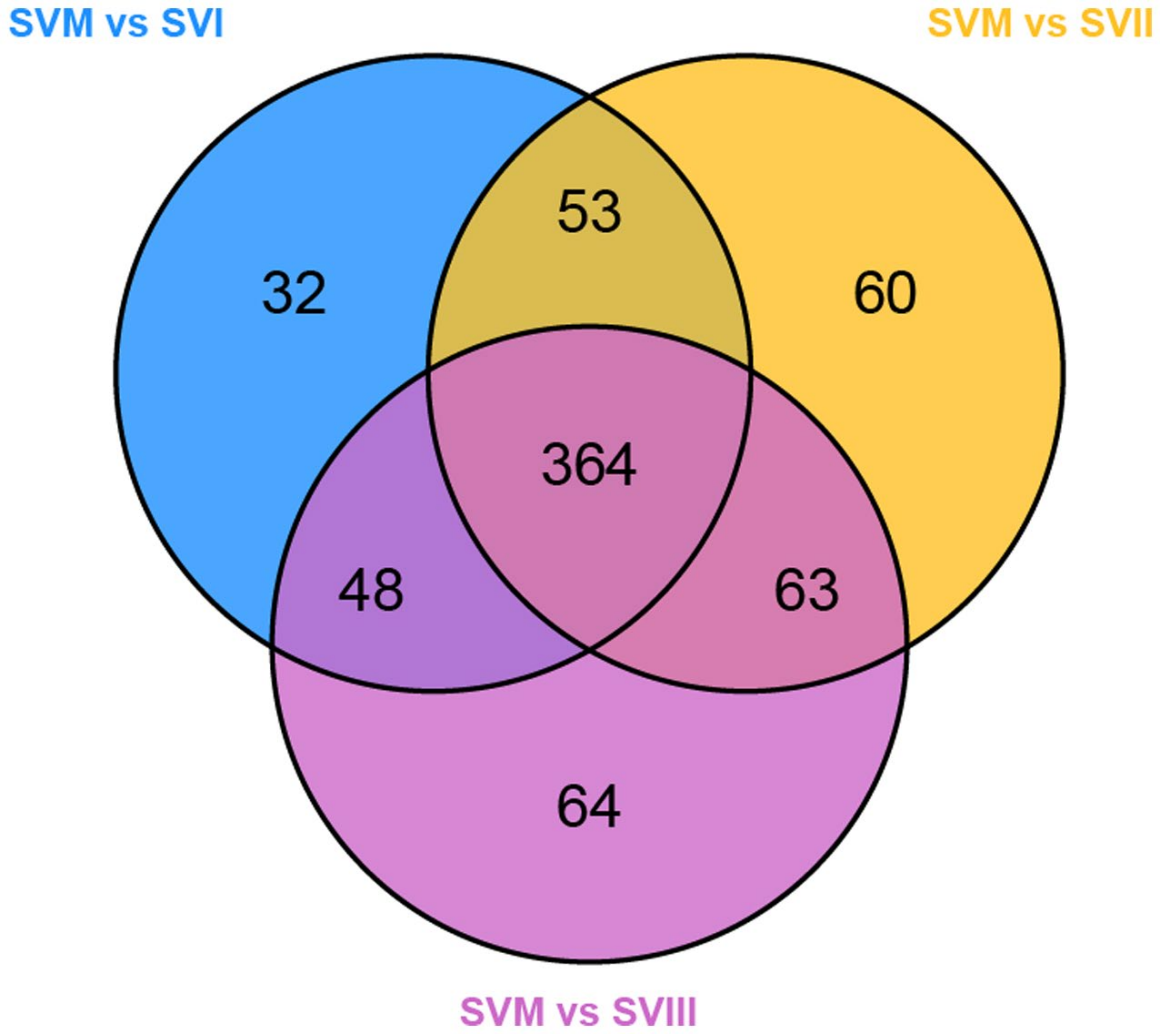
Venn diagram of differential metabolites of SVM-related pairwise comparison groups.

To identify the potential biomarkers of the mycelia and the fruiting bodies, we carried out pairwise comparisons between SVM and SVI, SVM and SVII, and SVM and SVIII. As a result, 854, 844, and 861 differential metabolites were identified in SVM and SVI, SVM and SVII, and SVM and SVIII comparison groups, respectively. A total of 841 differential metabolites were present in all three groups. Further analyses revealed that 364 out of the 841 differential metabolites were significantly different (≥2 fold) (Figure 5). Out of the 364 metabolites, 258 metabolites showed significantly higher content in SVM than in any SVF. The 258 metabolites were determined as the potential biomarkers in SVM, including 57 lipids, 49 amino acids and derivatives, 38 organic acids, 34 alkaloids, 31 nucleotides and derivatives, 18 others, 15 phenolic acids, 12 flavonoids, and 4 terpenoids. In addition to the 258 potential biomarkers of SVM, 99 metabolites showed significantly higher content in any SVF than in SVM. The 99 metabolites were identified as the potential biomarkers in SVF, including 30 phenolic acids, 28 flavonoids, 8 organic acids, 6 saccharides, 5 lipids, 4 terpenoids, 4 alkaloids, 4 amino acids and derivatives, 3 nucleotides and derivatives, 2 others, 1 quinone, 1 chromone, 1 alcohol compound, 1 lignan, and 1 coumarin.

**Figure 6:**
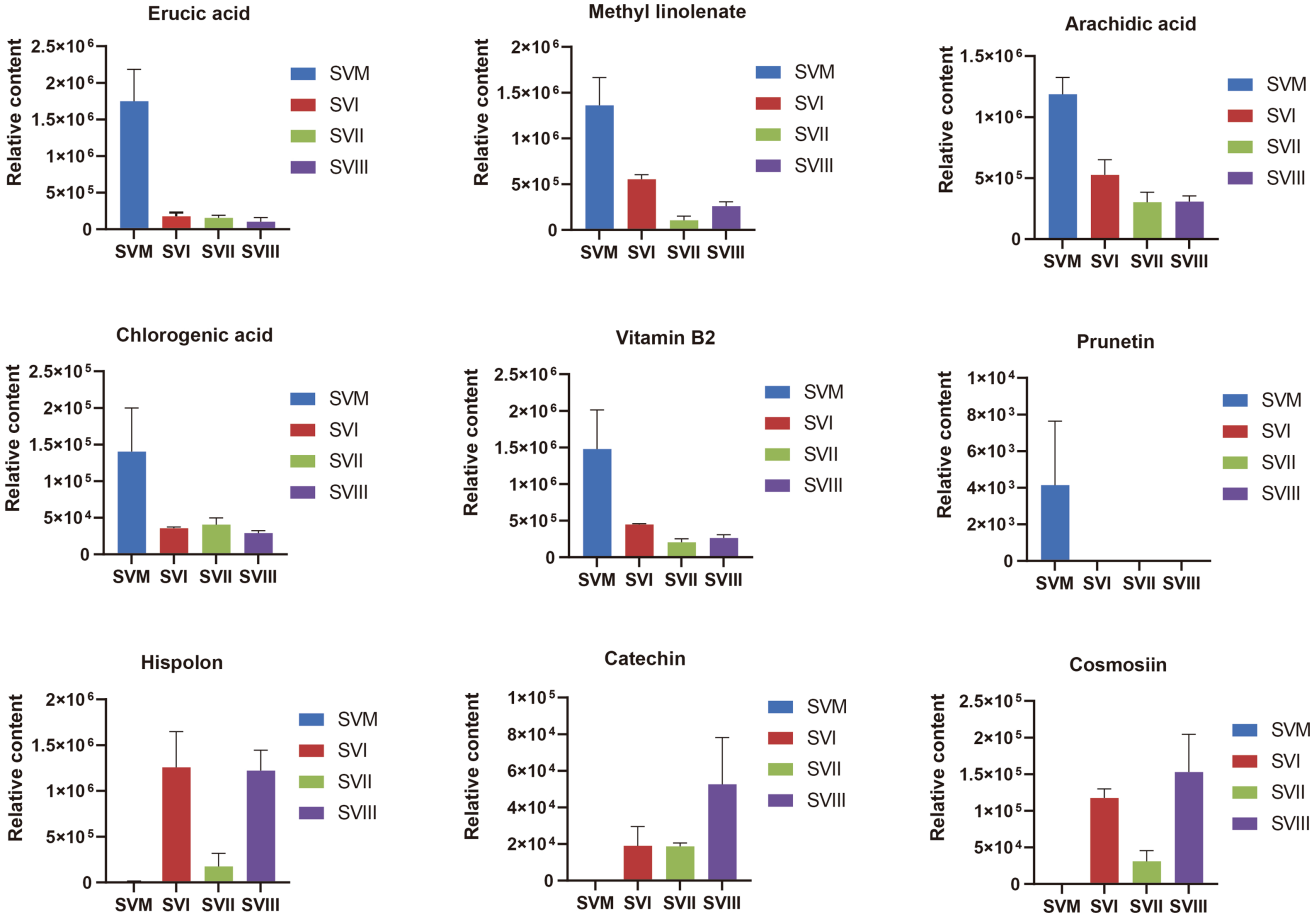
Differences in the relative content of nine iconic and crucial metabolites between the SVF and SVM. The y-axis shows the relative content of each metabolite, and the x-axis represents the four samples. The error bars represent the standard deviation.

Some of these potential biomarkers are determined as key active metabolites and active pharmacological metabolites associated with resistance to the six major diseases as mentioned above. These potential biomarkers are listed in Supplementary Table S7. More importantly, nine metabolites belonged to both the key active metabolites and active pharmacological metabolites associated with resistance to the six major diseases. The nine metabolites are considered to be the most prominent and crucial metabolites in the mycelia and fruiting bodies of *S. vaninii*. The relative content of the nine metabolites in the four samples of *S. vaninii* is shown in Figure 6.

#### 3.6.2 Identification of potential biomarkers of the fruiting bodies of 3 growth years of *Sanghuangporus vaninii*

**Figure 7:**
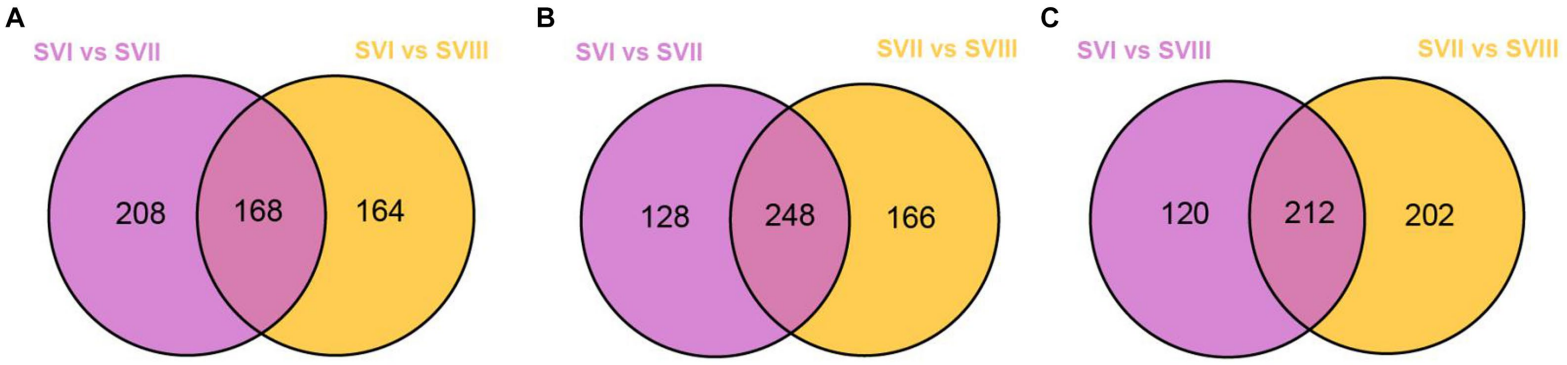
Venn diagrams of the differential metabolites of SVI-related, SVII-related, and SVIII-related comparison groups. **(A)** SVI-related groups; **(B)** SVII-related groups; **(C)** SVIII-related groups.

To identify the potential biomarkers of the fruiting bodies at three harvest stages, we conducted pairwise comparisons between SVI and SVII, SVI and SVIII, and SVII and SVIII. As a result, a total of 814, 826, and 818 differential metabolites were identified in SVI and SVII, SVI and SVIII, and SVII and SVIII, respectively. Out of them, a total of 807 differential metabolites were present in SVI-related comparison groups (SVI vs. SVII; SVI vs. SVIII), 799 differential metabolites were present in SVII-related comparison groups (SVI vs. SVII; SVII vs. SVIII), and 811 differential metabolites were present in SVIII-related comparison groups (SVI vs. SVIII; SVII vs. SVIII). Further analyses revealed that 168 differential metabolites were significantly different (≥2 fold) for SVI-related comparison groups (Figure 7A), 248 metabolites were significantly different (≥2 fold) for SVII-related comparison groups (Figure 7B), and 212 metabolites were significantly different (|log2(fold change)| ≥ 1) for SVIII-related comparison groups (Figure 7C). Out of these metabolites, 112 metabolites showed a significantly higher content (≥ 2 fold) in SVI, 55 metabolites showed a significantly higher content (≥ 2 fold) in SVII, and 79 metabolites showed a significantly higher content (≥ 2 fold) in SVIII. The 112, 55, and 79 metabolites were determined as the potential biomarkers in SVI, SVII, and SVIII, respectively.

**Figure 8:**
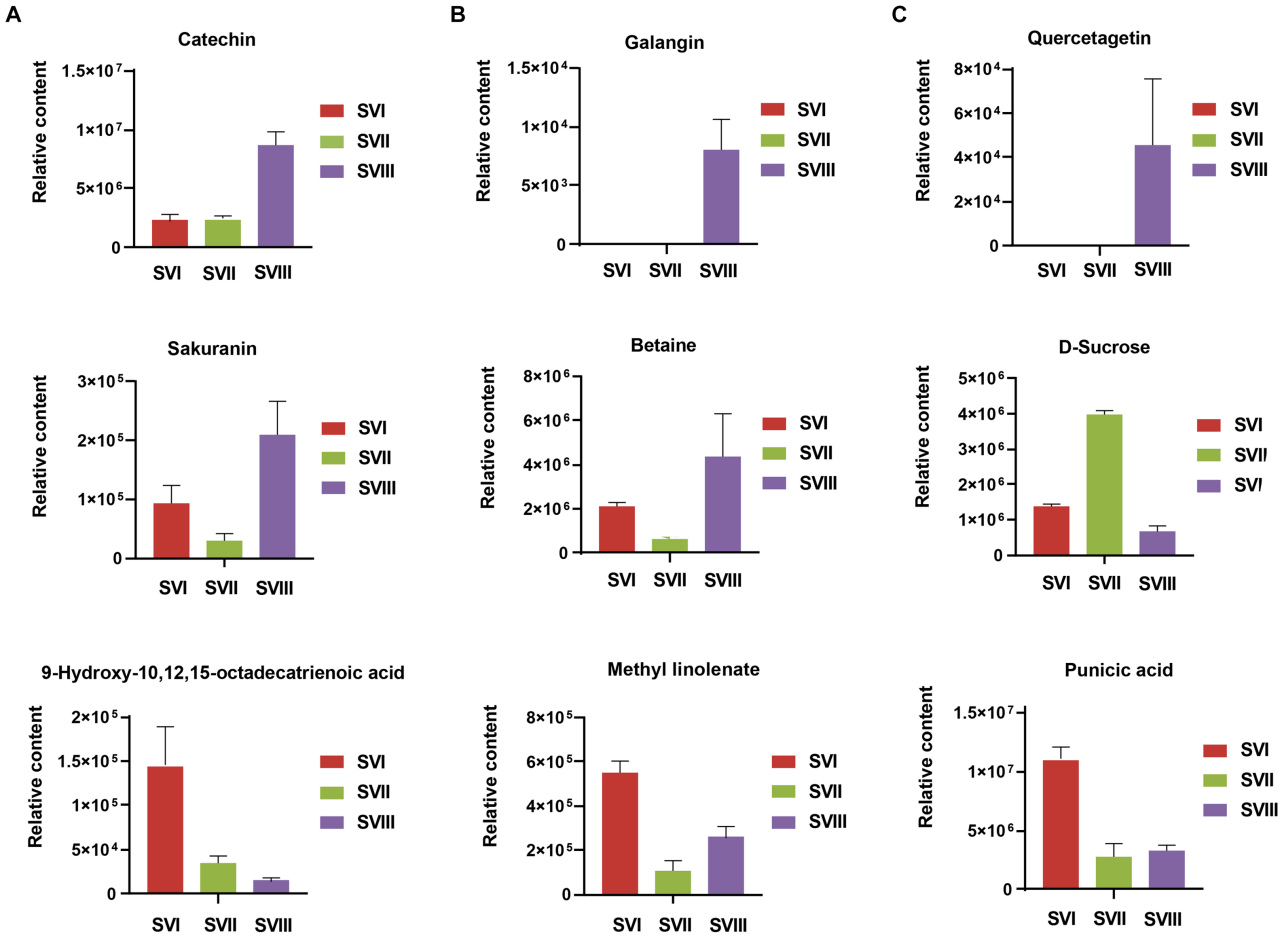
Differences of relative content of nine prominent and crucial metabolites between SVI, SVII, and SVIII. The y-axis shows the relative content of each metabolite, and the x-axis represents the three fruiting body samples. The error bars represent the standard deviation.

Some of these potential biomarkers are determined as the key active metabolites and active pharmacological metabolites associated with resistance to the six major diseases. The potential biomarkers are listed in Supplementary Table S8. More importantly, nine metabolites belonged to both the key active metabolites and active pharmacological metabolites for resistance to the six major diseases. The nine metabolites are considered to be the most prominent and crucial metabolites in the fruiting bodies of different growth years. The relative content of the nine metabolites in the three fruiting body samples of *S. vaninii* is shown in Figure 8.

## 4 Discussion

### 4.1 *Sanghuangporus vaninii* has great potential as an effective antitumor/cancer pharmaceutical

Despite a lengthy history of the usage of *S. vaninii* as a traditional Chinese medicine, it has not been listed in the Chinese Pharmacopoeia due to the lack of knowledge of its active metabolites. Metabolomics technologies were employed to comprehensively analyze the metabolites of four samples of *S. vaninii*. TCMSP was utilized to identify metabolites with beneficial health effects in these samples. A total of 81 key active metabolites and 157 active pharmaceutical metabolites associated with resistance to the six major diseases were identified, mainly involved in flavonoids, phenolic acids, terpenoids, and lipids. These active metabolites were associated with at least 174 diseases, with tumors/cancers being the most prevalent. This suggested that *S. vaninii* showed significant potential to become an excellent antitumor/cancer drug. This is further corroborated by the findings of a previous study. Wan et al. (2020) isolated four polysaccharide sub-fractions (SVP-W, SVP-1, SVP-2, and SVP-3) from the aqueous extract of *S. vaninii* and found that all inhibited the proliferation of non-small cell lung cancer cell lines A549, 95-D, and NCI-H460. Guo et al. (2022) confirmed that the 60% ethanol extract of *S. vaninii* inhibited effects on the proliferation of SW480 human colon cancer cells, inducing cell apoptosis and blocking G2/M period cells. Qiu et al. (2022) showed that inoscavin A exerted its antitumor activity in an HT-29 colon cancer cell in a xenograft mouse model. Cheng et al. (2023) isolated a biomacromolecule (SVPS2) from *S. vaninii* and demonstrated that it may be a promising therapeutic agent against colon cancer.

### 4.2 The mycelium of *Sanghuangporus vaninii* shows potential as a substitute for the fruiting bodies of *Sanghuangporus vaninii*

Due to the increased genetic stability and shorter production cycles of *S. vaninii*, we conducted a study to examine the metabolic differences between the mycelium and its fruiting bodies. Our goal was to determine whether the mycelia may serve as a substitute for the fruiting bodies. The number of metabolites in the mycelium and fruiting bodies of *S. vaninii* did not show significant differences, as shown in Figure 1A. Nevertheless, there were notable differences in the composition and concentration of metabolites in the mycelium and fruiting bodies of *S. vaninii*. Overall, the mycelium of *S. vaninii* had significantly higher accumulation levels of primary metabolites compared to the fruiting bodies of *S. vaninii*. These metabolites included lipids, amino acids and their derivatives, and nucleotides and their derivatives, as shown in Figures 4A–C. In contrast, the fruiting bodies exhibited higher accumulation levels of secondary metabolites, including phenolic acids and flavonoids, as seen in Figures 4A–C. The mycelium of *S. vaninii* contains some prominent and crucial metabolites (Supplementary Table S7), which may be the key to facilitating its substitution for the fruiting bodies. Furthermore, the mycelium of *S. vaninii* has a less complex composition of secondary metabolites, which enhances its use in the isolation and purification of a particular monomer molecule. This offers a theoretical foundation for the isolation and purification of a chemical with a biological activity.

### 4.3 It is crucial to determine the optimal harvesting stage for the fruiting bodies of *Sanghuangporus vaninii*

To examine the changes in metabolites of the fruiting bodies of *S. vaninii*, we conducted a study comparing the metabolic differences among SVI, SVII, and SVIII. The number and composition of metabolites were consistent across SVI, SVII, and SVIII (Figure 1B). Nevertheless, there were notable variations in the concentration of metabolites. Overall, SVI exhibited a higher concentration of primary metabolites such as lipids, organic acids, and amino acids and their derivatives, as well as nucleotides and their derivatives (Figures 4D,E). On the other hand, SVIII showed a higher concentration of secondary metabolites, specifically phenolic acids and flavonoids (Figures 4E,F). When considering the active metabolites, there were also notable variations among SVI, SVII, and SVIII (Supplementary Tables S7, S8). Despite having the shortest growth years, SVI possesses a number of active metabolites, including 9-hydroxy-10,12,15-octadecatrienoic acid, methyl linolenate, and punicic acid. This suggests that the growth years do not necessarily lead to an increase in the content of active metabolites in the fruiting bodies of *S. vaninii*. In order to optimize the utilization of *S. vaninii*, it is important to regulate the period of harvesting of the fruiting bodies of *S. vaninii* according to specific purposes.

## 5 Conclusion

To the best of our knowledge, this study is the first to focus on the identification of comprehensive active metabolites in *S. vaninii* and perform a comparison of metabolites between the mycelia and fruiting bodies of *S. vaninii* during the three different harvest stages. Using integrated metabolomics and TCMSP, 81 key active metabolites and 157 active pharmaceutical metabolites in *S. vaninii* associated with resistance to the six major diseases were preliminarily determined. This suggested that *S. vaninii* has significant potential to become an excellent drug, especially an antitumor/cancer drug. The results in metabolic differences among four samples of *S. vaninii* implied that mycelium has a certain degree of substitutability for the fruiting body of *S. vaninii*, and increasing growth years does not necessarily enhance the content. This present study provides a theoretical basis for the development and utilization of *S. vaninii*.

## Data availability statement

The original contributions presented in the study are included in the article/Supplementary material, further inquires can be directed to the corresponding author.

## Author contributions

YQ: Writing – original draft, Writing – review & editing, Software, Methodology, Formal analysis. X-YG: Writing – original draft, Software, Methodology, Formal analysis. X-YX: Writing – review & editing, Investigation, Formal analysis. J-XH: Writing – original draft, Visualization, Conceptualization. S-LL: Writing – original draft, Investigation, Data curation. H-BG: Writing – original draft, Resources, Project administration. A-GX: Writing – original draft, Funding acquisition, Conceptualization. R-HY: Writing – original draft, Resources, Data curation. X-DY: Writing – review & editing, Funding acquisition, Supervision, Conceptualization.

## Footnotes

^1^ http://www.shimadzu.com.cn/

^2^ https://www.thermofisher.cn/cn/zh/home/brands/applied-biosystems.html
