# Supplementary material for: Widely targeted metabolomics analysis of Sanghuangporus vaninii mycelia and fruiting bodies at different harvest stages
Source: Front Microbiol. 2024 May 23;15:1391558. doi: 10.3389/fmicb.2024.1391558 (PMC11153664; doi:10.3389/fmicb.2024.1391558)
Supplement: Supplementary file 1 [file Table_1.DOCX]

| Class | SVM | SVI | SVII | SVIII | Sum |
| --- | --- | --- | --- | --- | --- |
| Lipids | 138 | 137 | 124 | 124 | 143 |
| Organic acids | 89 | 105 | 93 | 106 | 113 |
| Phenolic acids | 102 | 107 | 96 | 103 | 112 |
| Amino acids and derivatives | 97 | 96 | 89 | 92 | 100 |
| Flavonoids | 65 | 77 | 74 | 65 | 97 |
| Saccharides | 73 | 73 | 64 | 70 | 78 |
| Alkaloids | 77 | 72 | 74 | 74 | 77 |
| Nucleotides and derivatives | 62 | 59 | 60 | 61 | 64 |
| Terpenoids | 30 | 31 | 31 | 31 | 37 |
| Lignans and coumarins | 10 | 12 | 13 | 11 | 14 |
| Vitamin | 11 | 11 | 11 | 11 | 11 |
| Others | 2 | 4 | 4 | 5 | 5 |
| Aldehyde compounds | 5 | 4 | 4 | 4 | 5 |
| Quinones | 3 | 3 | 3 | 3 | 3 |
| Alcohol compounds | 1 | 2 | 1 | 3 | 3 |
| Chromones | 1 | 2 | 2 | 2 | 2 |
| Sum | 766 | 795 | 743 | 765 | 864 |

**Supplementary Table 1.** Metabolites count on compound-class basis in four samples of *S. vaninii*.

**Supplementary Table 2.** Metabolites unique to the mycelium and fruiting bodies of *S. vaninii*.

| Class | SVM | SVI | SVII | SVIII |
| --- | --- | --- | --- | --- |
| Flavonoids (40) |  | 3'-O-Methyl-(-)-epicatechin | 3'-O-Methyl-(-)-epicatechin | 3'-O-Methyl-(-)-epicatechin |
|  |  | Isorhamnetin-3-O-rhamnoside | Isorhamnetin-3-O-rhamnoside | Isorhamnetin-3-O-rhamnoside |
|  |  | 6-C-Methylquercetin-3-O-rhamnoside | 6-C-Methylquercetin-3-O-rhamnoside | 6-C-Methylquercetin-3-O-rhamnoside |
|  |  | Dihydrokaempferide | Dihydrokaempferide | Dihydrokaempferide |
|  |  | 6-Methoxyquercetin-3-O-rhamnoside | 6-Methoxyquercetin-3-O-rhamnoside | 6-Methoxyquercetin-3-O-rhamnoside |
|  |  | Isohemiphloin | Isohemiphloin | Isohemiphloin |
|  |  | Luteolin-3'-O-glucoside | Luteolin-3'-O-glucoside | Luteolin-3'-O-glucoside |
|  |  | Quercetin-3-O-(6''-O-acetyl)glucoside | Quercetin-3-O-(6''-O-acetyl)glucoside | Quercetin-3-O-(6''-O-acetyl)glucoside |
|  |  | Tricin-4'-O-Benzoic acid | Tricin-4'-O-Benzoic acid | Tricin-4'-O-Benzoic acid |
|  |  | 2'-Hydroxy-3,4,5,3',4',6'-hexameth-oxychalcone | 2'-Hydroxy-3,4,5,3',4',6'-hexameth-oxychalcone | 2'-Hydroxy-3,4,5,3',4',6'-hexameth-oxychalcone |
|  |  | 8-Methoxykaempferol-7-O-rhamnoside | 8-Methoxykaempferol-7-O-rhamnoside | 8-Methoxykaempferol-7-O-rhamnoside |
|  |  | Naringenin-4'-O-glucoside | Naringenin-4'-O-glucoside | Naringenin-4'-O-glucoside |
|  |  | Ladanetin-6-O-β-D-glucosid | Ladanetin-6-O-β-D-glucosid | Ladanetin-6-O-β-D-glucosid |
|  |  | Yuanhuanin | Yuanhuanin | Yuanhuanin |
|  |  | Apigenin-4'-O-glucoside | Apigenin-4'-O-glucoside | Apigenin-4'-O-glucoside |
|  |  | Cosmosiin | Cosmosiin | Cosmosiin |
|  |  | Kaempferol-3-O-(2''-O-acetyl)glucuronide | Kaempferol-3-O-(2''-O-acetyl)glucuronide | Kaempferol-3-O-(2''-O-acetyl)glucuronide |
|  |  | Galangin-7-O-glucoside | Galangin-7-O-glucoside | Galangin-7-O-glucoside |
|  |  | Dihydrokaempferol-7-O-glucoside | Dihydrokaempferol-7-O-glucoside | Dihydrokaempferol-7-O-glucoside |
|  |  | Dihydrokaempferol-3-O-glucoside | Dihydrokaempferol-3-O-glucoside | Dihydrokaempferol-3-O-glucoside |
|  |  | Eriodictyol-3'-O-glucoside | Eriodictyol-3'-O-glucoside | Eriodictyol-3'-O-glucoside |
|  |  | 1,3,6,7-Tetrahydroxyxanthone | 1,3,6,7-Tetrahydroxyxanthone | 1,3,6,7-Tetrahydroxyxanthone |
|  |  | Chrysoeriol-5-O-glucoside | Chrysoeriol-5-O-glucoside | Chrysoeriol-5-O-glucoside |
|  |  | Isosalipurposide | Isosalipurposide | Isosalipurposide |
|  |  | Quercetin-7-O-glucoside | Quercetin-7-O-glucoside | Quercetin-7-O-glucoside |
|  |  | 2,6,7,4'-Tetrahydroxyisoflavanone | 2,6,7,4'-Tetrahydroxyisoflavanone | 2,6,7,4'-Tetrahydroxyisoflavanone |
|  |  | Quercetin-4'-O-glucoside | Quercetin-4'-O-glucoside | Quercetin-4'-O-glucoside |
|  |  | Hesperetin-5-O-glucoside | Hesperetin-5-O-glucoside | Hesperetin-5-O-glucoside |
|  |  | Catechin | Catechin | Catechin |
|  | 7-O-Methylnaringenin |  |  |  |
|  | Sakuranetin |  |  |  |
|  | Prunetin |  |  |  |
|  | Rhamnocitrin |  |  |  |
|  | Hispidulin |  |  |  |
|  | 6,7,8-Tetrahydroxy-5-methoxyflavone |  |  |  |
|  | Eriocitrin |  |  |  |
|  | Lonicerin |  |  |  |
|  | Naringenin chalcone |  |  |  |
|  | Butin |  |  |  |
|  | Pinobanksin |  |  |  |
| Phenolic acids (20) |  | 3-(3-Hydroxyphenyl)-propionic acid | 3-(3-Hydroxyphenyl)-propionic acid | 3-(3-Hydroxyphenyl)-propionic acid |
|  |  | 3-Hydroxyphenylacetic acid Methyl Ester | 3-Hydroxyphenylacetic acid Methyl Ester | 3-Hydroxyphenylacetic acid Methyl Ester |
|  |  | Protocatechuic acid ethyl ester | Protocatechuic acid ethyl ester | Protocatechuic acid ethyl ester |
|  |  | 4-Nitrocatechol | 4-Nitrocatechol | 4-Nitrocatechol |
|  |  | 1,2-O-Diferuloylglycerol | 1,2-O-Diferuloylglycerol | 1,2-O-Diferuloylglycerol |
|  |  | 1,3-O-Diferuloylglycerol | 1,3-O-Diferuloylglycerol | 1,3-O-Diferuloylglycerol |
|  |  | Methyl ferulate | Methyl ferulate | Methyl ferulate |
|  |  | 2,5-Dihydroxyacetophenone | 2,5-Dihydroxyacetophenone | 2,5-Dihydroxyacetophenone |
|  |  | Pyrocatechol | Pyrocatechol | Pyrocatechol |
|  |  | Gallein | Gallein | Gallein |
|  |  | 1-O-p-Hydroxycinnamoyl-3-O-caffeoylglycerol | 1-O-p-Hydroxycinnamoyl-3-O-caffeoylglycerol | 1-O-p-Hydroxycinnamoyl-3-O-caffeoylglycerol |
|  |  | isoamericanin A | isoamericanin A | isoamericanin A |
|  |  | 2-O-Salicyl-6-O-Galloyl-D-Glucose | 2-O-Salicyl-6-O-Galloyl-D-Glucose | 2-O-Salicyl-6-O-Galloyl-D-Glucose |
|  |  | (S)-2-Hydroxy-3-(4-Hydroxyphenyl)Propanoic acid | (S)-2-Hydroxy-3-(4-Hydroxyphenyl)Propanoic acid | (S)-2-Hydroxy-3-(4-Hydroxyphenyl)Propanoic acid |
|  |  | Hydroxytyrosol | Hydroxytyrosol | Hydroxytyrosol |
|  |  | 5-(2-Hydroxyethyl)-2-O-glucosylphenol | 5-(2-Hydroxyethyl)-2-O-glucosylphenol | 5-(2-Hydroxyethyl)-2-O-glucosylphenol |
|  |  | 3,4-dihydroxy-allylbenzene-3-O-β-D-glucopyranoside | 3,4-dihydroxy-allylbenzene-3-O-β-D-glucopyranoside | 3,4-dihydroxy-allylbenzene-3-O-β-D-glucopyranoside |
|  |  | Phelligridin J | Phelligridin J | Phelligridin J |
|  | 3,4-Dimethoxycinnamic acid |  |  |  |
|  | Ethyl caffeate |  |  |  |
| Lipids (9) |  | LysoPC 20:4 | LysoPC 20:4 | LysoPC 20:4 |
|  |  | Myristic acid | Myristic acid | Myristic acid |
|  |  | 3-Dehydrosphinganine | 3-Dehydrosphinganine | 3-Dehydrosphinganine |
|  |  | Arachidonic acid | Arachidonic acid | Arachidonic acid |
|  | Octanoic acid |  |  |  |
|  | 10,16-Dihydroxypalmitic acid |  |  |  |
|  | 1-α-Linolenoyl-glycerol |  |  |  |
|  | LysoPE 17:1 |  |  |  |
|  | Heptadecanoic acid |  |  |  |
| Organic acids (8) |  | 3-Amino-1-propionic sulfonic acid | 3-Amino-1-propionic sulfonic acid | 3-Amino-1-propionic sulfonic acid |
|  |  | L-Lactic acid | L-Lactic acid | L-Lactic acid |
|  |  | Allantoin | Allantoin | Allantoin |
|  |  | Tartronate semialdehyde | Tartronate semialdehyde | Tartronate semialdehyde |
|  |  | Hydroxypyruvic acid | Hydroxypyruvic acid | Hydroxypyruvic acid |
|  |  | Methanesulfonic acid | Methanesulfonic acid | Methanesulfonic acid |
|  | Triethyl citrate |  |  |  |
|  | Mevalonic acid |  |  |  |
| Alkaloids (7) |  | Feruloylcholine | Feruloylcholine | Feruloylcholine |
|  |  | Nicotinic acid N-oxide | Nicotinic acid N-oxide | Nicotinic acid N-oxide |
|  |  | p-Coumaroylcadaverine | p-Coumaroylcadaverine | p-Coumaroylcadaverine |
|  |  | N,N-Dimethylformamide | N,N-Dimethylformamide | N,N-Dimethylformamide |
|  | 1-Acetyl-β-carboline |  |  |  |
|  | 2-Aminophenol |  |  |  |
|  | Isobutyryl carnitine |  |  |  |
| Others (7) |  | 2-(2'-hydroxypropyl)-5-methyl-7-hydroxychromone | 2-(2'-hydroxypropyl)-5-methyl-7-hydroxychromone | 2-(2'-hydroxypropyl)-5-methyl-7-hydroxychromone |
|  |  | 2-(Dodecylamino)-3-phenyl-1-propanol | 2-(Dodecylamino)-3-phenyl-1-propanol | 2-(Dodecylamino)-3-phenyl-1-propanol |
|  |  | 6-hydroxycoumarin | 6-hydroxycoumarin | 6-hydroxycoumarin |
|  |  | 1,7-Bis(4-hydroxy-3-methoxyphenyl)heptane-3,5-diol | 1,7-Bis(4-hydroxy-3-methoxyphenyl)heptane-3,5-diol | 1,7-Bis(4-hydroxy-3-methoxyphenyl)heptane-3,5-diol |
|  | Mesitaldehyde |  |  |  |
|  | D-Glucose 6-phosphate |  |  |  |
|  | D-Fructose 6-Phosphate |  |  |  |
| Terpenoids (7) |  | Asiatic acid | Asiatic acid | Asiatic acid |
|  |  | Limonin | Limonin | Limonin |
|  |  | (13α,14β,17S)-16β-Hydroxy-3-oxo-5α-lanosta-7,24-diene-21-oic acid | (13α,14β,17S)-16β-Hydroxy-3-oxo-5α-lanosta-7,24-diene-21-oic acid | (13α,14β,17S)-16β-Hydroxy-3-oxo-5α-lanosta-7,24-diene-21-oic acid |
|  | Hederagenin |  |  |  |
|  | Pomolic acid |  |  |  |
|  | Alphitolic acid |  |  |  |
|  | 2-Hydroxyoleanolic acid |  |  |  |
| Amino acids and derivatives (4) |  | L-Homomethionine | L-Homomethionine | L-Homomethionine |
|  |  | L-Serine | L-Serine | L-Serine |
|  |  | L-Glutamic acid | L-Glutamic acid | L-Glutamic acid |
|  | Cyclo(Ser-Pro) |  |  |  |
| Nucleotides and derivatives (4) |  | N6-Isopentenyladenine | N6-Isopentenyladenine | N6-Isopentenyladenine |
|  |  | Cytidylic acid | Cytidylic acid | Cytidylic acid |
|  | Cordycepin |  |  |  |
|  | N6-methyladenosine |  |  |  |
| Lignans and Coumarins (3) |  | erythro-guaiacylglycerol-β-coniferyl ether | erythro-guaiacylglycerol-β-coniferyl ether | erythro-guaiacylglycerol-β-coniferyl ether |
|  |  | Pinoresinol-4-O-glucoside | Pinoresinol-4-O-glucoside | Pinoresinol-4-O-glucoside |
|  |  | Coumarin-3-carboxylic acid | Coumarin-3-carboxylic acid | Coumarin-3-carboxylic acid |

**Supplementary Table 3.** Metabolites unique to the fruiting bodies at three different harvest stages of *S. vaninii*, respectively.

| Class | SVI | SVII | SVIII |
| --- | --- | --- | --- |
| Flavonoids  (10) |  |  | Myricetin-3,7,3'-trimethyl ether |
|  |  |  | Galangin |
|  |  |  | Eriodictyol |
|  |  |  | 3-O-Acetylpinobanksin |
|  |  |  | Quercetagetin |
|  |  |  | Kaempferol-3-O-neohesperidoside |
|  |  | Kaempferol-3-O-rhamnoside |  |
|  |  | Kaempferol-7-O-rhamnoside |  |
|  | Aromadendrin-7-O-glucoside |  |  |
|  | Isovitexin-7-O-xyloside-2''-O-rhamnoside |  |  |
| Organic acids (5) |  |  | Citraconic acid |
|  |  |  | Benzoylformic acid |
|  | Creatine |  |  |
|  | Oxaloacetic acid |  |  |
|  | Methyl jasmonate |  |  |
| Phenolic acids (7) |  |  | Salicylaldehyde |
|  |  |  | 3-Aminosalicylic acid |
|  |  |  | 2',4'-Dihydroxyacetophenone |
|  |  | 4-O-Methylgallic acid |  |
|  |  | Coniferyl alcohol |  |
|  | Vnilloyltartaric acid |  |  |
|  | p-Coumaric acid ethyl ester |  |  |
| Lipids  (6) |  | LysoPE 18:4 |  |
|  | 12-Oxo-phytodienoic acid |  |  |
|  | 12-Hydroxydodecanoic acid |  |  |
|  | 13-Hydroxy-9Z,11E-octadecadienoic acid |  |  |
|  | Crepenynic acid |  |  |
|  | PA(18:2/0:0) |  |  |
| Amino acids and derivatives  (6) |  |  | N-acetyl-beta-alanine |
|  |  | Trimethyllysine |  |
|  |  | N-Acetyl-L-methionine |  |
|  | N-Alpha-Acetyl-L-Asparagine |  |  |
|  | 5-Oxo-L-Proline |  |  |
|  | L-Lysine |  |  |
| Alkaloids  (5) |  |  | N-benzoyl-2-aminoethyl-β-D-glucopyranoside |
|  |  |  | Dioxindole-3-acetic acid |
|  | 3-Hydroxypyridine |  |  |
|  | Methyl L-pyroglutamate |  |  |
|  | Putrescine |  |  |
| Others  (3) |  |  | Manninotriose |
|  |  |  | Noreugenin |
|  |  |  | 3-hydroxy-5-methoxybibenzyl |
| Nucleotides and derivatives  (2) |  |  | N7-Methylguanosine |
|  |  | 1-Methyladenine |  |
| Coumarin  (1) |  |  | 6-MethylCoumarin |

**Supplementary Table 4.** A list of 81 key active metabolites that identified in *S. vaninii*.

| Class | Metabolites | Related target numbers | Related diseases numbers | Oral bioavailability (OB %) | Drug-likeness (DL) |
| --- | --- | --- | --- | --- | --- |
| Flavonoids  (30) | Rhamnocitrin | 19 | 106 | 12.9 | 0.27 |
|  | 3,5,6,7,8,3',4'-Heptamethoxyflavone | 81 | 81 | 23.91 | 0.58 |
|  | Cosmosiin | 2 | 32 | 9.68 | 0.74 |
|  | Butin | 7 | 48 | 69.94 | 0.21 |
|  | Catechin | 11 | 57 | 54.83 | 0.24 |
|  | Dihydrokaempferide | N/A | N/A | 23.1 | 0.27 |
|  | Diosmin | 10 | 50 | 12.7 | 0.66 |
|  | Eriodictyol | 9 | 47 | 71.79 | 0.24 |
|  | Galangin | 19 | 95 | 45.55 | 0.21 |
|  | Gancaonin G | 20 | 112 | 60.44 | 0.39 |
|  | Hesperetin-5-O-glucoside | N/A | N/A | 21.82 | 0.83 |
|  | Hesperetin-7-O-glucoside | N/A | N/A | 7.69 | 0.82 |
|  | Hesperetin-7-O-neohesperidoside | N/A | N/A | 11.57 | 0.69 |
|  | Hesperetin-7-O-rutinoside | 6 | 33 | 13.33 | 0.67 |
|  | Hispidulin | 17 | 75 | 30.97 | 0.27 |
|  | Isohyperoside | 1 | 8 | 8.31 | 0.77 |
|  | Narcissin | 1 | 8 | 5.09 | 0.65 |
|  | Isosinensetin | 27 | 126 | 51.15 | 0.44 |
|  | Luteolin-7,3'-di-O-glucoside | N/A | 33 | 13.42 | 0.62 |
|  | Prunin | 2 | 32 | 9.33 | 0.74 |
|  | Naringin | 5 | 20 | 6.92 | 0.78 |
|  | Narirutin | 1 | 8 | 8.15 | 0.75 |
|  | Nobiletin | 35 | 118 | 61.67 | 0.52 |
|  | Prunetin | 22 | 108 | 5.41 | 0.24 |
|  | Quercetagetin | 8 | 24 | 45.01 | 0.31 |
|  | Hyperin | 8 | 77 | 6.94 | 0.77 |
|  | Sakuranin | 3 | 12 | 17.04 | 0.78 |
|  | Sinensetin | 21 | 100 | 50.56 | 0.45 |
|  | Tangeretin | 29 | 118 | 21.38 | 0.43 |
|  | Astilbin | 5 | 29 | 40.54 | 0.74 |
| Lipids  (18) | Vaccenic acid | 2 | 2 | 33.13 | 0.14 |
|  | 1-Eicosanol | N/A | N/A | 12.1 | 0.16 |
|  | 1-Linoleoylglycerol | 1 | 2 | 37.18 | 0.3 |
|  | 9-Hydroxy-10,12,15-octadecatrienoic acid | 2 | 34 | 37.92 | 0.17 |
|  | Arachidic acid | 8 | 49 | 16.66 | 0.19 |
|  | Arachidonic acid | 4 | 34 | 45.57 | 0.2 |
|  | Docosanoic acid | N/A | N/A | 15.69 | 0.26 |
|  | Eicosenoic acid | 2 | 2 | 28.64 | 0.2 |
|  | Elaidic acid | 24 | 85 | 33.13 | 0.14 |
|  | Erucic acid | 15 | 71 | 28.56 | 0.26 |
|  | Hexadecanedioic acid | N/A | N/A | 20.72 | 0.16 |
|  | Linoleic acid | 16 | 40 | 41.9 | 0.14 |
|  | Methyl linolenate | 6 | 35 | 46.15 | 0.17 |
|  | Petroselinic acid | 2 | 2 | 33.13 | 0.14 |
|  | Punicic acid | 3 | 34 | 44.9 | 0.15 |
|  | Ricinoleic acid | 3 | 34 | 32.57 | 0.16 |
|  | Tetracosanoic acid | 2 | N/A | 14.9 | 0.33 |
|  | γ-Linolenic acid | 6 | 41 | 45.01 | 0.15 |
| Terpenoids  (15) | 2-Hydroxyoleanolic acid | N/A | N/A | 17.38 | 0.74 |
|  | 3-Epiursolic acid | N/A | N/A | 17.52 | 0.76 |
|  | Alphitolic acid | N/A | N/A | 16.59 | 0.76 |
|  | Asiatic acid | N/A | N/A | 16.69 | 0.72 |
|  | Betulin | N/A | N/A | 15.48 | 0.78 |
|  | Betulinic acid | N/A | N/A | 55.38 | 0.78 |
|  | Dehydroabietic acid | 21 | 92 | 14.93 | 0.28 |
|  | Hederagenin | N/A | N/A | 22.42 | 0.74 |
|  | Isomangiferolic acid | N/A | N/A | 16.85 | 0.73 |
|  | Limonin | N/A | N/A | 21.3 | 0.57 |
|  | Lupenone | N/A | N/A | 11.66 | 0.78 |
|  | Madasiatic acid | N/A | N/A | 18.42 | 0.72 |
|  | Mangiferolic acid | N/A | N/A | 36.16 | 0.84 |
|  | Pomolic acid | N/A | N/A | 16.85 | 0.73 |
|  | Ursolic acid | 55 | 98 | 16.77 | 0.75 |
| Phenolic acids  (7) | Bis(2-ethylhexyl)phthalate | 4 | 6 | 43.59 | 0.35 |
|  | Chlorogenic acid | 1 | 32 | 11.93 | 0.33 |
|  | Cryptochlorogenic acid | N/A | N/A | 24.5 | 0.33 |
|  | Diisooctyl Phthalate | 21 | 21 | 43.59 | 0.39 |
|  | Usnic acid | 2 | 39 | 5.36 | 0.39 |
|  | Hispidin | N/A | N/A | N/A | N/A |
|  | Hispolon | N/A | N/A | N/A | N/A |
| Nucleotides and derivatives  (4) | Adenosine | N/A | N/A | 18.06 | 0.18 |
|  | Guanosine | N/A | N/A | 21.43 | 0.21 |
|  | Inosine | N/A | N/A | 11.17 | 0.18 |
|  | Uridine 5'-monophosphate | 1 | 3 | 40.25 | 0.2 |
| Vitamins  (2) | Phylloquinone (Vitamin K1) | 1 | 32 | 47.6 | 0.66 |
|  | Vitamin B2 | 2 | 39 | 6.79 | 0.5 |
| Saccharides  (3) | D-Sucrose | 30 | 30 | 7.17 | 0.23 |
|  | Planteose | N/A | N/A | 12.15 | 0.66 |
|  | Raffinose | 4 | 7 | 11.79 | 0.66 |
| Alkaloid (1) | Betaine | 4 | 37 | 24.8 | 0.55 |
| Lignan (1) | Trachelogenin | N/A | N/A | 9.37 | 0.47 |

**Supplementary Table 5.** A list of 157 active pharmaceutical metabolites for six major diseases*-*resistance that identified in *S. vaninii*.

| Class | Anti-cancer/tumor ingredient | Anti-cardiovascular ingredient | Anti-asthma disease's ingredients | Anti-diabetic ingredient | Anti-parkinson's ingredients | Anti-alzheimer's ingredients |
| --- | --- | --- | --- | --- | --- | --- |
| Phenolic acids (46) |  |  |  |  | Anthranilic Acid |  |
|  |  | Syringic acid |  |  |  |  |
|  | 2-Phenylethanol |  |  |  |  |  |
|  |  |  |  |  | 4-Hydroxybenzoic acid |  |
|  | Hydroquinone | Hydroquinone | Hydroquinone | Hydroquinone |  |  |
|  | Rosmarinic acid | Rosmarinic acid |  |  |  |  |
|  | Hydrocinnamic acid | Hydrocinnamic acid |  | Hydrocinnamic acid | Hydrocinnamic acid | Hydrocinnamic acid |
|  | Tyrosol; 4-Hydroxyphenylethanol | Tyrosol; 4-Hydroxyphenylethanol | Tyrosol; 4-Hydroxyphenylethanol |  | Tyrosol; 4-Hydroxyphenylethanol |  |
|  |  | 2,6-Di-tert-butylphenol | 2,6-Di-tert-butylphenol |  | 2,6-Di-tert-butylphenol | 2,6-Di-tert-butylphenol |
|  |  | Butyl isobutyl phthalate | Butyl isobutyl phthalate | Butyl isobutyl phthalate | Butyl isobutyl phthalate | Butyl isobutyl phthalate |
|  | 4-O-Methylgallic Acid | 4-O-Methylgallic Acid | 4-O-Methylgallic Acid |  | 4-O-Methylgallic Acid | 4-O-Methylgallic Acid |
|  | 2,4-Dihydroxybenzoic acid | 2,4-Dihydroxybenzoic acid | 2,4-Dihydroxybenzoic acid |  | 2,4-Dihydroxybenzoic acid | 2,4-Dihydroxybenzoic acid |
|  | Dimethyl phthalate | Dimethyl phthalate | Dimethyl phthalate |  |  | Dimethyl phthalate |
|  | p-Coumaric acid | p-Coumaric acid | p-Coumaric acid |  | p-Coumaric acid | p-Coumaric acid |
|  | 3-Hydroxy-4-methoxybenzoic acid; Isovanillic Acid | 3-Hydroxy-4-methoxybenzoic acid; Isovanillic Acid | 3-Hydroxy-4-methoxybenzoic acid; Isovanillic Acid |  | 3-Hydroxy-4-methoxybenzoic acid; Isovanillic Acid | 3-Hydroxy-4-methoxybenzoic acid; Isovanillic Acid |
|  | 2',4'-Dihydroxyacetophenone | 2',4'-Dihydroxyacetophenone | 2',4'-Dihydroxyacetophenone |  | 2',4'-Dihydroxyacetophenone | 2',4'-Dihydroxyacetophenone |
|  | Isovanillin | Isovanillin | Isovanillin |  | Isovanillin | Isovanillin |
|  | Methyl gallate | Methyl gallate | Methyl gallate |  | Methyl gallate | Methyl gallate |
|  | 2-Hydroxycinnamic acid | 2-Hydroxycinnamic acid | 2-Hydroxycinnamic acid |  | 2-Hydroxycinnamic acid | 2-Hydroxycinnamic acid |
|  | Protocatechuic Acid Methyl Ester | Protocatechuic Acid Methyl Ester | Protocatechuic Acid Methyl Ester |  | Protocatechuic Acid Methyl Ester | Protocatechuic Acid Methyl Ester |
|  | Vanillic acid | Vanillic acid | Vanillic acid |  | Vanillic acid | Vanillic acid |
|  | Benzoic acid | Benzoic acid | Benzoic acid |  | Benzoic acid | Benzoic acid |
|  | Phthalic acid | Phthalic acid | Phthalic acid |  | Phthalic acid | Phthalic acid |
|  | 4-Hydroxyphenylacetic acid | 4-Hydroxyphenylacetic acid | 4-Hydroxyphenylacetic acid |  | 4-Hydroxyphenylacetic acid | 4-Hydroxyphenylacetic acid |
|  | Diisobutyl phthalate | Diisobutyl phthalate | Diisobutyl phthalate | Diisobutyl phthalate | Diisobutyl phthalate | Diisobutyl phthalate |
|  | Vanillin; 4-Hydroxy-3-Methoxybenzaldehyde | Vanillin; 4-Hydroxy-3-Methoxybenzaldehyde | Vanillin; 4-Hydroxy-3-Methoxybenzaldehyde |  | Vanillin; 4-Hydroxy-3-Methoxybenzaldehyde | Vanillin; 4-Hydroxy-3-Methoxybenzaldehyde |
|  | Cinnamic acid | Cinnamic acid | Cinnamic acid | Cinnamic acid | Cinnamic acid | Cinnamic acid |
|  | Ethyl caffeate | Ethyl caffeate | Ethyl caffeate |  | Ethyl caffeate | Ethyl caffeate |
|  | 3,4-Dihydroxybenzoic Acid Ethyl Ester (Protocatechuic acid ethyl ester) | 3,4-Dihydroxybenzoic Acid Ethyl Ester (Protocatechuic acid ethyl ester) | 3,4-Dihydroxybenzoic Acid Ethyl Ester (Protocatechuic acid ethyl ester) |  | 3,4-Dihydroxybenzoic Acid Ethyl Ester (Protocatechuic acid ethyl ester) | 3,4-Dihydroxybenzoic Acid Ethyl Ester (Protocatechuic acid ethyl ester) |
|  | 4-Methoxycinnamic acid | 4-Methoxycinnamic acid | 4-Methoxycinnamic acid |  | 4-Methoxycinnamic acid | 4-Methoxycinnamic acid |
|  | Homogentisic acid | Homogentisic acid | Homogentisic acid |  | Homogentisic acid | Homogentisic acid |
|  | Caffeic acid | Caffeic acid | Caffeic acid |  | Caffeic acid | Caffeic acid |
|  | Vanillin acetate | Vanillin acetate | Vanillin acetate |  | Vanillin acetate | Vanillin acetate |
|  | Syringaldehyde; 4-Hydroxy-3,5-Dimethoxybenzaldehyde | Syringaldehyde; 4-Hydroxy-3,5-Dimethoxybenzaldehyde | Syringaldehyde; 4-Hydroxy-3,5-Dimethoxybenzaldehyde |  | Syringaldehyde; 4-Hydroxy-3,5-Dimethoxybenzaldehyde | Syringaldehyde; 4-Hydroxy-3,5-Dimethoxybenzaldehyde |
|  | 3,4-Dihydroxybenzoic acid (Protocatechuic acid) | 3,4-Dihydroxybenzoic acid (Protocatechuic acid) | 3,4-Dihydroxybenzoic acid (Protocatechuic acid) | 3,4-Dihydroxybenzoic acid (Protocatechuic acid) | 3,4-Dihydroxybenzoic acid (Protocatechuic acid) | 3,4-Dihydroxybenzoic acid (Protocatechuic acid) |
|  | Hydroxytyrosol | Hydroxytyrosol | Hydroxytyrosol |  |  | Hydroxytyrosol |
|  | Isoferulic Acid | Isoferulic Acid | Isoferulic Acid |  |  | Isoferulic Acid |
|  | Ferulic acid | Ferulic acid | Ferulic acid |  |  | Ferulic acid |
|  | Coniferyl alcohol | Coniferyl alcohol | Coniferyl alcohol |  |  | Coniferyl alcohol |
|  | 3-(3-Hydroxyphenyl)-propionic acid | 3-(3-Hydroxyphenyl)-propionic acid | 3-(3-Hydroxyphenyl)-propionic acid | 3-(3-Hydroxyphenyl)-propionic acid | 3-(3-Hydroxyphenyl)-propionic acid | 3-(3-Hydroxyphenyl)-propionic acid |
|  | Salicylic acid | Salicylic acid | Salicylic acid |  |  | Salicylic acid |
|  | Dibutyl phthalate | Dibutyl phthalate | Dibutyl phthalate | Dibutyl phthalate | Dibutyl phthalate | Dibutyl phthalate |
|  |  | Bis(2-ethylhexyl)phthalate |  |  |  |  |
|  |  | Diisooctyl Phthalate | Diisooctyl Phthalate |  |  |  |
|  | Chlorogenic acid (3-O-Caffeoylquinic acid) | Chlorogenic acid (3-O-Caffeoylquinic acid) | Chlorogenic acid (3-O-Caffeoylquinic acid) |  |  | Chlorogenic acid (3-O-Caffeoylquinic acid) |
|  | Usnic acid | Usnic acid | Usnic acid |  |  | Usnic acid |
|  | Hispidin | Hispidin | Hispidin | Hispidin | Hispidin | Hispidin |
|  | Hispolon | Hispolon | Hispolon | Hispolon | Hispolon | Hispolon |
| Flavonoids (30) | Quercetin-3-O-sophoroside (Baimaside) | Quercetin-3-O-sophoroside (Baimaside) |  |  |  |  |
|  | Quercetin-3-O-glucoside (Isoquercitrin) | Quercetin-3-O-glucoside (Isoquercitrin) |  | Quercetin-3-O-glucoside (Isoquercitrin) |  |  |
|  | Kaempferol-7-O-rhamnoside | Kaempferol-7-O-rhamnoside | Kaempferol-7-O-rhamnoside |  |  | Kaempferol-7-O-rhamnoside |
|  | Kaempferol-3-O-rhamnoside (Afzelin)(Kaempferin) | Kaempferol-3-O-rhamnoside (Afzelin)(Kaempferin) | Kaempferol-3-O-rhamnoside (Afzelin)(Kaempferin) |  |  | Kaempferol-3-O-rhamnoside (Afzelin)(Kaempferin) |
|  | Quercetin-3-O-rhamnoside(Quercitrin) | Quercetin-3-O-rhamnoside(Quercitrin) | Quercetin-3-O-rhamnoside(Quercitrin) | Quercetin-3-O-rhamnoside(Quercitrin) |  | Quercetin-3-O-rhamnoside(Quercitrin) |
|  | Isohyperoside |  |  |  |  |  |
|  | Naringenin-7-O-Rutinoside(Narirutin) |  |  |  |  |  |
|  | Isorhamnetin-3-O-rutinoside (Narcissin) |  |  |  |  |  |
|  | Sakuranin | Sakuranin |  |  |  |  |
|  | Sinensetin (5,6,7,3',4'-pentamethoxyflavone) | Sinensetin (5,6,7,3',4'-pentamethoxyflavone) | Sinensetin (5,6,7,3',4'-pentamethoxyflavone) | Sinensetin (5,6,7,3',4'-pentamethoxyflavone) | Sinensetin (5,6,7,3',4'-pentamethoxyflavone) | Sinensetin (5,6,7,3',4'-pentamethoxyflavone) |
|  | Naringenin-7-O-Neohesperidoside(Naringin) | Naringenin-7-O-Neohesperidoside(Naringin) | Naringenin-7-O-Neohesperidoside(Naringin) | Naringenin-7-O-Neohesperidoside(Naringin) |  |  |
|  | Quercetagetin; 3,3',4',5,6,7-Hexahydroxyflavone | Quercetagetin; 3,3',4',5,6,7-Hexahydroxyflavone | Quercetagetin; 3,3',4',5,6,7-Hexahydroxyflavone | Quercetagetin; 3,3',4',5,6,7-Hexahydroxyflavone |  | Quercetagetin; 3,3',4',5,6,7-Hexahydroxyflavone |
|  | Taxifolin-3-O-rhamnoside (Astilbin) | Taxifolin-3-O-rhamnoside (Astilbin) |  |  |  |  |
|  | Apigenin-7-O-glucoside(Cosmosiin) | Apigenin-7-O-glucoside(Cosmosiin) | Apigenin-7-O-glucoside(Cosmosiin) |  |  | Apigenin-7-O-glucoside(Cosmosiin) |
|  | Naringenin-7-O-glucoside (Prunin) | Naringenin-7-O-glucoside (Prunin) | Naringenin-7-O-glucoside (Prunin) |  |  | Naringenin-7-O-glucoside (Prunin) |
|  | Luteolin-7,3'-di-O-glucoside | Luteolin-7,3'-di-O-glucoside | Luteolin-7,3'-di-O-glucoside |  |  | Luteolin-7,3'-di-O-glucoside |
|  | Hesperetin-7-O-rutinoside (Hesperidin) | Hesperetin-7-O-rutinoside (Hesperidin) | Hesperetin-7-O-rutinoside (Hesperidin) |  |  | Hesperetin-7-O-rutinoside (Hesperidin) |
|  | Eriodictyol (5,7,3',4'-Tetrahydroxyflavanone) | Eriodictyol (5,7,3',4'-Tetrahydroxyflavanone) | Eriodictyol (5,7,3',4'-Tetrahydroxyflavanone) |  |  | Eriodictyol (5,7,3',4'-Tetrahydroxyflavanone) |
|  | Diosmetin-7-O-rutinoside (Diosmin) | Diosmetin-7-O-rutinoside (Diosmin) | Diosmetin-7-O-rutinoside (Diosmin) | Diosmetin-7-O-rutinoside (Diosmin) |  | Diosmetin-7-O-rutinoside (Diosmin) |
|  | Quercetin-3-O-galactoside (Hyperin) | Quercetin-3-O-galactoside (Hyperin) | Quercetin-3-O-galactoside (Hyperin) | Quercetin-3-O-galactoside (Hyperin) |  | Quercetin-3-O-galactoside (Hyperin) |
|  | Catechin | Catechin | Catechin |  |  | Catechin |
|  | Hispidulin (5,7,4'-Trihydroxy-6-methoxyflavone) | Hispidulin (5,7,4'-Trihydroxy-6-methoxyflavone) | Hispidulin (5,7,4'-Trihydroxy-6-methoxyflavone) | Hispidulin (5,7,4'-Trihydroxy-6-methoxyflavone) | Hispidulin (5,7,4'-Trihydroxy-6-methoxyflavone) | Hispidulin (5,7,4'-Trihydroxy-6-methoxyflavone) |
|  | 3,5,6,7,8,3',4'-Heptamethoxyflavone | 3,5,6,7,8,3',4'-Heptamethoxyflavone | 3,5,6,7,8,3',4'-Heptamethoxyflavone | 3,5,6,7,8,3',4'-Heptamethoxyflavone |  | 3,5,6,7,8,3',4'-Heptamethoxyflavone |
|  | Galangin (3,5,7-Trihydroxyflavone) | Galangin (3,5,7-Trihydroxyflavone) | Galangin (3,5,7-Trihydroxyflavone) | Galangin (3,5,7-Trihydroxyflavone) |  | Galangin (3,5,7-Trihydroxyflavone) |
|  | 3,5,4'-Trihydroxy-7-methoxyflavone (Rhamnocitrin) | 3,5,4'-Trihydroxy-7-methoxyflavone (Rhamnocitrin) | 3,5,4'-Trihydroxy-7-methoxyflavone (Rhamnocitrin) | 3,5,4'-Trihydroxy-7-methoxyflavone (Rhamnocitrin) |  | 3,5,4'-Trihydroxy-7-methoxyflavone (Rhamnocitrin) |
|  | Prunetin (5,4'-Dihydroxy-7-methoxyisoflavone) | Prunetin (5,4'-Dihydroxy-7-methoxyisoflavone) | Prunetin (5,4'-Dihydroxy-7-methoxyisoflavone) | Prunetin (5,4'-Dihydroxy-7-methoxyisoflavone) |  | Prunetin (5,4'-Dihydroxy-7-methoxyisoflavone) |
|  | Gancaonin G | Gancaonin G | Gancaonin G | Gancaonin G |  | Gancaonin G |
|  | Nobiletin (5,6,7,8,3',4'-Hexamethoxyflavone) | Nobiletin (5,6,7,8,3',4'-Hexamethoxyflavone) | Nobiletin (5,6,7,8,3',4'-Hexamethoxyflavone) | Nobiletin (5,6,7,8,3',4'-Hexamethoxyflavone) |  | Nobiletin (5,6,7,8,3',4'-Hexamethoxyflavone) |
|  | Tangeretin (4',5,6,7,8-Pentamethoxyflavone) | Tangeretin (4',5,6,7,8-Pentamethoxyflavone) | Tangeretin (4',5,6,7,8-Pentamethoxyflavone) | Tangeretin (4',5,6,7,8-Pentamethoxyflavone) | Tangeretin (4',5,6,7,8-Pentamethoxyflavone) | Tangeretin (4',5,6,7,8-Pentamethoxyflavone) |
|  | Isosinensetin | Isosinensetin | Isosinensetin | Isosinensetin | Isosinensetin |  |
| Organic acids (20) | L-Pipecolic Acid |  |  |  |  | L-Pipecolic Acid |
|  | Creatine |  |  | Creatine |  | Creatine |
|  | cis-Citral | cis-Citral | cis-Citral | cis-Citral |  |  |
|  | Malonic acid | Malonic acid |  |  |  | Malonic acid |
|  | γ-Aminobutyric acid |  |  |  |  | γ-Aminobutyric acid |
|  | Allantoin | Allantoin | Allantoin |  |  | Allantoin |
|  | Quinic Acid | Quinic Acid | Quinic Acid |  |  | Quinic Acid |
|  | L-Malic acid |  | L-Malic acid | L-Malic acid |  | L-Malic acid |
|  | Piperonylic acid | Piperonylic acid | Piperonylic acid |  | Piperonylic acid | Piperonylic acid |
|  | Succinic acid | Succinic acid | Succinic acid | Succinic acid |  | Succinic acid |
|  | Pyruvic acid | Pyruvic acid | Pyruvic acid | Pyruvic acid |  | Pyruvic acid |
|  | D-(-)-Mandelic acid | D-(-)-Mandelic acid | D-(-)-Mandelic acid | D-(-)-Mandelic acid | D-(-)-Mandelic acid | D-(-)-Mandelic acid |
|  | 4-Guanidinobutyric acid | 4-Guanidinobutyric acid | 4-Guanidinobutyric acid | 4-Guanidinobutyric acid |  | 4-Guanidinobutyric acid |
|  | 2-Hydroxyphenylacetic acid | 2-Hydroxyphenylacetic acid | 2-Hydroxyphenylacetic acid |  |  | 2-Hydroxyphenylacetic acid |
|  | Citric Acid | Citric Acid | Citric Acid | Citric Acid |  | Citric Acid |
|  | Fumaric acid | Fumaric acid | Fumaric acid | Fumaric acid |  | Fumaric acid |
|  | L-Lactic Acid | L-Lactic Acid | L-Lactic Acid | L-Lactic Acid |  | L-Lactic Acid |
|  | Phenylpyruvic acid | Phenylpyruvic acid | Phenylpyruvic acid | Phenylpyruvic acid | Phenylpyruvic acid | Phenylpyruvic acid |
|  | DL-3-Phenyllactic acid | DL-3-Phenyllactic acid | DL-3-Phenyllactic acid | DL-3-Phenyllactic acid | DL-3-Phenyllactic acid | DL-3-Phenyllactic acid |
|  | Oxalic acid | Oxalic acid | Oxalic acid | Oxalic acid |  | Oxalic acid |
| Lipids  (21) |  | Heptadecanoic acid |  |  |  |  |
|  |  | Tridecanoic Acid |  |  |  |  |
|  | Palmitoleic Acid | Palmitoleic Acid | Palmitoleic Acid |  |  | Palmitoleic Acid |
|  | (7Z)-Hexadecenoic acid | (7Z)-Hexadecenoic acid | (7Z)-Hexadecenoic acid |  |  | (7Z)-Hexadecenoic acid |
|  | Pentadecanoic Acid | Pentadecanoic Acid | Pentadecanoic Acid |  |  | Pentadecanoic Acid |
|  | Myristic Acid | Myristic Acid | Myristic Acid |  |  | Myristic Acid |
|  | Palmitic acid | Palmitic acid | Palmitic acid | Palmitic acid |  | Palmitic acid |
|  |  | 1-Linoleoylglycerol |  |  |  |  |
|  |  | Petroselinic acid |  |  |  |  |
|  |  | 11-Octadecanoic acid(Vaccenic acid) |  |  |  |  |
|  |  | Eicosenoic acid |  |  |  |  |
|  | Punicic acid (9Z,11E,13Z-octadecatrienoic acid) | Punicic acid (9Z,11E,13Z-octadecatrienoic acid) | Punicic acid (9Z,11E,13Z-octadecatrienoic acid) |  |  | Punicic acid (9Z,11E,13Z-octadecatrienoic acid) |
|  | 9-Hydroxy-10,12,15-octadecatrienoic acid | 9-Hydroxy-10,12,15-octadecatrienoic acid | 9-Hydroxy-10,12,15-octadecatrienoic acid |  |  | 9-Hydroxy-10,12,15-octadecatrienoic acid |
|  | Ricinoleic acid | Ricinoleic acid | Ricinoleic acid |  |  | Ricinoleic acid |
|  | Methyl linolenate | Methyl linolenate | Methyl linolenate |  |  | Methyl linolenate |
|  | Linoleic acid | Linoleic acid | Linoleic acid | Linoleic acid |  | Linoleic acid |
|  | Arachidonic Acid | Arachidonic Acid | Arachidonic Acid |  |  | Arachidonic Acid |
|  | γ-Linolenic Acid | γ-Linolenic Acid | γ-Linolenic Acid |  |  | γ-Linolenic Acid |
|  | Arachidic acid | Arachidic acid | Arachidic acid | Arachidic acid |  | Arachidic acid |
|  | Erucic acid | Erucic acid | Erucic acid | Erucic acid | Erucic acid | Erucic acid |
|  | Elaidic Acid | Elaidic Acid | Elaidic Acid | Elaidic Acid | Elaidic Acid | Elaidic Acid |
| Alkaloids  (9) |  |  |  |  | Candicine |  |
|  |  |  |  |  |  | Stachydrine |
|  | Putrescine | Putrescine | Putrescine | Putrescine |  |  |
|  |  | L-Tyramine | L-Tyramine |  | L-Tyramine | L-Tyramine |
|  | Trigonelline | Trigonelline | Trigonelline |  |  | Trigonelline |
|  | Indole-3-carboxylic acid | Indole-3-carboxylic acid | Indole-3-carboxylic acid |  | Indole-3-carboxylic acid | Indole-3-carboxylic acid |
|  | Histamine | Histamine | Histamine |  |  | Histamine |
|  | Tryptamine | Tryptamine | Tryptamine |  |  | Tryptamine |
|  | Betaine | Betaine | Betaine |  |  | Betaine |
| Amino acids and derivatives (8) | L-Lysine |  |  |  |  |  |
|  |  | L-Leucine | L-Leucine |  |  |  |
|  | L-Isoleucine | L-Isoleucine |  |  |  | L-Isoleucine |
|  | L-Serine | L-Serine |  | L-Serine |  | L-Serine |
|  | L-Proline | L-Proline | L-Proline |  |  | L-Proline |
|  | L-Aspartic Acid | L-Aspartic Acid | L-Aspartic Acid | L-Aspartic Acid |  | L-Aspartic Acid |
|  | 3,4-Dihydroxy-L-phenylalanine (L-Dopa) | 3,4-Dihydroxy-L-phenylalanine (L-Dopa) | 3,4-Dihydroxy-L-phenylalanine (L-Dopa) |  |  | 3,4-Dihydroxy-L-phenylalanine (L-Dopa) |
|  | L-Citrulline | L-Citrulline | L-Citrulline |  |  | L-Citrulline |
| Nucleotides and derivatives  (6) | Adenine | Adenine |  |  |  |  |
|  | Hypoxanthine | Hypoxanthine |  |  |  |  |
|  | Xanthine | Xanthine |  |  |  |  |
|  | Guanine | Guanine |  |  |  |  |
|  | Uracil |  |  |  | Uracil |  |
|  | Uridine 5'-monophosphate |  |  |  |  |  |
| Vitamins  (5) | Nicotinamide | Nicotinamide | Nicotinamide |  |  | Nicotinamide |
|  | Nicotinic acid (Vitamin B3) | Nicotinic acid (Vitamin B3) | Nicotinic acid (Vitamin B3) |  |  | Nicotinic acid (Vitamin B3) |
|  | Riboflavin (Vitamin B2) | Riboflavin (Vitamin B2) | Riboflavin (Vitamin B2) |  |  | Riboflavin (Vitamin B2) |
|  | Pyridoxine | Pyridoxine | Pyridoxine |  | Pyridoxine | Pyridoxine |
|  | Phylloquinone (Vitamin K1) | Phylloquinone (Vitamin K1) | Phylloquinone (Vitamin K1) |  |  | Phylloquinone (Vitamin K1) |
| Saccharides  (4) | D-Galacturonic acid | D-Galacturonic acid | D-Galacturonic acid |  |  | D-Galacturonic acid |
|  | 1,5-Anhydro-D-glucitol | 1,5-Anhydro-D-glucitol | 1,5-Anhydro-D-glucitol |  |  | 1,5-Anhydro-D-glucitol |
|  | Raffinose |  |  |  |  |  |
|  | D-Sucrose | D-Sucrose | D-Sucrose | D-Sucrose | D-Sucrose |  |
| Terpenoids (2) | Dehydroabietic acid | Dehydroabietic acid | Dehydroabietic acid | Dehydroabietic acid | Dehydroabietic acid | Dehydroabietic acid |
|  | Ursolic acid | Ursolic acid | Ursolic acid | Ursolic acid |  | Ursolic acid |
| Lignans and Coumarins  (2) | Esculetin (6,7-Dihydroxycoumarin) | Esculetin (6,7-Dihydroxycoumarin) | Esculetin (6,7-Dihydroxycoumarin) | Esculetin (6,7-Dihydroxycoumarin) |  |  |
|  | Isoscopoletin (6-Hydroxy-7-Methoxycoumarin) | Isoscopoletin (6-Hydroxy-7-Methoxycoumarin) | Isoscopoletin (6-Hydroxy-7-Methoxycoumarin) |  |  | Isoscopoletin (6-Hydroxy-7-Methoxycoumarin) |
| Quinone  (1) | Laccaic acid D | Laccaic acid D | Laccaic acid D |  |  | Laccaic acid D |
| Alcohol compound (1) | Eucommiol | Eucommiol | Eucommiol |  |  | Eucommiol |

**Supplementary Table 6.** The number of differential metabolites based on compound-class in each pairwise comparsion group.

| Metabolite class |  | SVI-SVII | SVI-SVIII | SVII-SVIII | SVM-SVI | SVM-SVII | SVM-VIII |
| --- | --- | --- | --- | --- | --- | --- | --- |
| Amino acids and derivatives | up | 11 | 4 | 24 | 7 | 6 | 5 |
|  | down | 41 | 38 | 28 | 65 | 75 | 61 |
| Phenolic acids | up | 8 | 29 | 50 | 41 | 32 | 44 |
|  | down | 31 | 9 | 5 | 22 | 32 | 26 |
| Nucleotides and derivatives | up | 6 | 2 | 21 | 3 | 4 | 3 |
|  | down | 31 | 3 | 16 | 42 | 47 | 46 |
| Flavonoids | up | 5 | 32 | 35 | 36 | 28 | 39 |
|  | down | 24 | 68 | 17 | 18 | 19 | 25 |
| Quinones | up | 0 | 0 | 1 | 1 | 1 | 1 |
|  | down | 0 | 0 | 0 | 0 | 0 | 0 |
| Lignans and Coumarins | up | 1 | 5 | 7 | 5 | 3 | 3 |
|  | down | 6 | 10 | 3 | 1 | 3 | 2 |
| Alcohol compounds | up | 0 | 1 | 2 | 0 | 0 | 1 |
|  | down | 1 | 2 | 0 | 5 | 7 | 0 |
| Others | up | 0 | 1 | 0 | 14 | 11 | 3 |
|  | down | 2 | 0 | 1 | 17 | 21 | 1 |
| Aldehyde compounds | up | 0 | 0 | 3 | 2 | 1 | 0 |
|  | down | 1 | 1 | 0 | 0 | 0 | 4 |
| Chromone | up | 0 | 1 | 1 | 0 | 0 | 2 |
|  | down | 1 | 1 | 0 | 3 | 3 | 0 |
| Saccharides | up | 18 | 0 | 10 | 2 | 2 | 9 |
|  | down | 13 | 0 | 22 | 0 | 1 | 22 |
| Vitamin | up | 0 | 2 | 3 | 1 | 1 | 0 |
|  | down | 5 | 17 | 2 | 0 | 1 | 5 |
| Alkaloids | up | 8 | 11 | 17 | 10 | 6 | 7 |
|  | down | 29 | 26 | 21 | 39 | 46 | 44 |
| Terpenoids | up | 1 | 5 | 11 | 10 | 4 | 14 |
|  | down | 12 | 5 | 4 | 4 | 8 | 10 |
| Organic acids | up | 8 | 5 | 32 | 22 | 10 | 15 |
|  | down | 43 | 2 | 17 | 45 | 57 | 51 |
| Lipids | up | 9 | 4 | 36 | 12 | 9 | 9 |
|  | down | 61 | 48 | 25 | 70 | 102 | 85 |
| sum | up | 75 | 102 | 253 | 166 | 118 | 156 |
|  | down | 301 | 230 | 161 | 331 | 422 | 383 |
|  | sum | 376 | 332 | 414 | 497 | 540 | 539 |

**Supplementary Table 7.** Potential biomarkers that belonged to the key active metabolites and active pharmacological metabolites for six major diseases-resistance in the mycelium and fruiting bodies of *S. vaninii*, respectively.

|  | Class | Identification of key active metabolites of Traditional Chinese Medicines in *S. vaninii* | Identification of active pharmaceutical metabolites for six major diseases-resistance in *S. vaninii* |
| --- | --- | --- | --- |
| SVM  (38) | Lipids | Lignoceric acid |  |
|  |  | Behenic acid |  |
|  |  |  | Octanoic acid |
|  |  |  | Heptadecanoic acid |
|  |  |  | (7Z)-Hexadecenoic acid |
|  |  | Erucic acid | Erucic acid |
|  |  | Methyl linolenate | Methyl linolenate |
|  |  | Arachidic acid | Arachidic acid |
|  | Amino acids and derivatives |  | L-Dopa |
|  |  |  | L-Proline |
|  |  |  | L-Leucine |
|  |  |  | L-Isoleucine |
|  | Phenolic acids |  | Ethyl caffeate |
|  |  |  | Cinnamic acid |
|  |  |  | p-Coumaric acid |
|  |  |  | 2-Hydroxycinnamic acid |
|  |  |  | 4-Hydroxybenzoic acid |
|  |  | Chlorogenic acid | Chlorogenic acid |
|  | Alkaloids |  | Trigonelline |
|  |  |  | Putrescine |
|  |  |  | Histamine |
|  |  |  | Indole-3-carboxylic acid |
|  |  | 2-Aminophenol |  |
|  | Organic acids |  | Phenylpyruvic acid |
|  |  |  | DL-3-Phenyllactic acid |
|  |  |  | L-Malic acid |
|  |  |  | Citric acid |
|  |  |  | L-Pipecolic acid |
|  | Nucleotides and derivatives | Adenosine |  |
|  |  | Guanosine |  |
|  |  |  | Hypoxanthine |
|  |  |  | Xanthine |
|  | Flavonoids |  | Baimaside |
|  |  | Prunetin | Prunetin |
|  | Terpenoids | Alphitolic acid |  |
|  |  | 2-Hydroxyoleanolic acid |  |
|  | Vitamins |  | Pyridoxine |
|  |  | Vitamin B2 | Vitamin B2 |
| SVF  (22) | Phenolic acids |  | Phelligridin A |
|  |  |  | Phelligridin B |
|  |  |  | Phelligridin J |
|  |  | Hispolon | Hispolon |
|  |  |  | 2,4-Dihydroxybenzoic acid |
|  |  |  | Tyrosol |
|  |  |  | Hydroxytyrosol |
|  |  |  | Protocatechuic acid |
|  |  |  | Homogentisic acid |
|  |  |  | Protocatechuic acid Methyl Ester |
|  |  |  | 3-(3-Hydroxyphenyl)-propionic acid |
|  |  |  | Salicylic acid |
|  |  |  | Protocatechuic acid ethyl ester |
|  | Flavonoids | Catechin | Catechin |
|  |  | Cosmosiin | Cosmosiin |
|  |  |  | Hesperetin-5-O-glucoside |
|  | Organic acids |  | Malonic acid |
|  |  |  | Allantoin |
|  | Lipids |  | Arachidonic acid |
|  |  |  | Myristic acid |
|  | Amino acids and derivative |  | L-Serine |
|  | Quinone |  | Laccaic acid D |

**Supplementary Table 8.** Potential biomarkers that belonged to the key active metabolites and active pharmacological metabolites for six major diseases-resistance in the fruiting bodies of *S. vaninii* at three different harvest stages, respectively.

|  | Class | Identification of key active ingredients of Traditional Chinese Medicines in *S. vaninii* | Identification of active pharmaceutical ingredients for six major diseases-resistance in *S. vaninii* |
| --- | --- | --- | --- |
| SVIII (18) | Phenolic acids |  | 2',4'-Dihydroxyacetophenone |
|  |  |  | 4-Hydroxybenzoic acid |
|  |  |  | Rosmarinic acid |
|  |  |  | Salicylic acid |
|  |  |  | Syringic acid |
|  |  |  | Tyrosol |
|  |  |  | Vanillic acid |
|  | Flavonoids | Catechin | Catechin |
|  |  | Galangin | Galangin |
|  |  | Quercetagetin | Quercetagetin |
|  |  | Sakuranin | Sakuranin |
|  | Terpenoids | Isomangiferolic acid |  |
|  |  | Lupenone |  |
|  | Organic acids |  | Allantoin |
|  |  |  | DL-3-Phenyllactic acid |
|  | Alkaloid | Betaine | Betaine |
|  | Quinone |  | Laccaic acid D |
| SVII  (7) | Saccharide |  | Planteose |
|  | Phenolic acids |  | 4-O-Methylgallic acid |
|  |  |  | Ferulic acid |
|  | Flavonoids |  | Kaempferol-3-O-rhamnoside |
|  |  |  | Kaempferol-7-O-rhamnoside |
|  | Amino acids and derivative |  | L-Aspartic acid |
|  | Organic acid |  | Quinic acid |
| SVI  (17) | Lipids | 9-Hydroxy-10,12,15-octadecatrienoic acid | 9-Hydroxy-10,12,15-octadecatrienoic acid |
|  |  | Methyl linolenate | Methyl linolenate |
|  |  | Punicic acid | Punicic acid |
|  | Organic acids |  | Creatine |
|  |  |  | Succinic acid |
|  |  |  | γ-Aminobutyric acid |
|  | Alkaloids |  | L-Tyramine |
|  |  |  | Putrescine |
|  |  |  | Tryptamine |
|  | Phenolic acids |  | 2-Phenylethanol |
|  |  |  | Caffeic acid |
|  | Nucleotides and derivatives | Adenosine |  |
|  |  |  | Hypoxanthine |
|  | Amino acids and derivative |  | L-Serine |
|  | Coumarin |  | Esculetin |
|  | Vitamin |  | Pyridoxine |
|  | Flavonoid |  | Quercetin-3-O-glucoside |
